# Body Mass Index and the Risk of Hypertension-Diabetes Comorbidity in Elderly Population: A Prospective Cohort in China

**DOI:** 10.5334/gh.1487

**Published:** 2025-10-16

**Authors:** Haimeng Zhang, Guangling Li, Fan Li, Jiangang Jiang

**Affiliations:** 1 Nanchang University, Jiangxi, 330031, China; 2 Division of Cardiology, Department of Internal Medicine, Tongji Hospital, Tongji Medical College, Huazhong University of Science and Technology, Wuhan 430030, China; 3 Hubei Key Laboratory of Genetics and Molecular Mechanisms of Cardiological Disorders, Wuhan 430030, China

**Keywords:** hypertension, diabetes mellitus, comorbidity, Body Mass Index, obesity, hypertension-diabetes comorbidity

## Abstract

**Background:**

Body mass index (BMI) is closely linked to hypertension and diabetes mellitus (DM). However, the association between BMI and hypertension-diabetes comorbidity in elderly population of China remains uncertain.

**Methods:**

This cohort study was conducted based on a prospective database of Chinese Longitudinal Healthy Longevity Survey (CLHLS). The primary outcome was hypertension-diabetes comorbidity. The secondary outcomes included hypertension and DM. BMI was categorized into four groups according to Chinese guidelines: underweight (BMI < 18.5 kg/m^2^), normal weight (BMI 18.5–23.9 kg/m^2^), overweight (BMI 24.0–27.9 kg/m^2^), and obesity (BMI ≥ 28.0 kg/m^2^).

**Results:**

The final analysis included 5,342 individuals for hypertension, 6,335 for DM, and 6,414 for hypertension-diabetes comorbidity (all individuals aged 65 years and above). Cox regression analysis of the hypertension-diabetes comorbidity revealed the adjusted hazard ratio (HR) for the underweight group was 0.747 (95% confidence interval [CI], 0.651–0.857), for the overweight group was 1.517 (95% CI, 1.309–1.758), and for the obesity group was 1.620 (95% CI, 1.237–2.121) comparing with normal weight group (reference). When considering BMI as a continuous variable, the adjusted HR was 1.043 (95% CI, 1.029–1.059). Employing a multi-model adjusting strategy based on the directed acyclic graph, the HR for individuals with BMI ≥ 24.0 (versus BMI < 24.0) was 1.486 (95% CI, 1.301–1.698). Restricted cubic splines indicate a positive linear trend between BMI (range in BMI ≥ 24) and the risk of hypertension-diabetes comorbidity. The relationship between BMI and secondary outcomes exhibited results similar to those of the primary outcome.

Additionally, compared to the Han ethnic, other ethnic had a significantly lower risk of the primary outcome, with an adjusted HR of 0.334 (95% CI, 0.235–0.475). Similar findings were observed for the secondary outcomes.

**Conclusions:**

Increased BMI is significantly associated with a higher risk of hypertension-diabetes comorbidity, hypertension, and DM in elderly population of China. Additionally, Han participants (versus non-Han) have a notably greater risk of developing hypertension-diabetes comorbidity, hypertension, and DM. Greater attention should be paid to obesity in elderly Han Chinese individuals, given its significant associated disease burden.

## Introduction

Hypertension and diabetes mellitus (DM) are two of the most prevalent chronic diseases worldwide, with their incidence showing a steady upward trend in recent years (1,2,3,4,5). Body Mass Index (BMI) has emerged as a significant risk factor for both hypertension and DM (6,7,8,9,10,11). Hypertension and DM frequently coexist, with shared mechanisms like insulin resistance, inflammatory responses, and oxidative stress (12,13,14,15,16,17). Patients with hypertension-diabetes comorbidity often face a heightened cardiovascular risk, necessitating more stringent management and treatment. While prior studies have examined BMI’s association with either hypertension or DM, few have specifically examined its association with hypertension-diabetes comorbidity in elderly populations and those available are limited to retrospective or cross-sectional designs (18,19). Most existing research on BMI and cardiometabolic diseases has focused on middle-aged adults. However, aging alters body composition, metabolic regulation, and disease susceptibility, making it unclear whether BMI thresholds for risk prediction in younger cohorts apply to the elderly.

This study utilizes large-scale prospective data from elderly population of China to examine BMI as both a categorical and continuous variable, comprehensively exploring its associations with hypertension-diabetes comorbidity, hypertension, and DM from multiple perspectives, employing comprehensive covariate adjustment strategies to ensure robust effect estimation. By elucidating these relationships, we aim to provide a scientific foundation for healthcare policymakers in weight management of elderly population.

## Methods

### Study populations

The study employed data from the Chinese Longitudinal Healthy Longevity Survey (CLHLS) database, which is the largest sample of elderly individuals in China, conducted across 23 provinces, municipalities, and autonomous regions. Participants completed face-to-face questionnaires, underwent anthropometric assessments, and provided biological samples for laboratory testing. This study was approved by the Peking University Medical Ethics Committee (IRB 00001052–13074). All participants or their legal guardians provided written informed consent at the beginning of the survey.

Consistent with the observational nature of large-scale real-world studies, we implemented pragmatic screening criteria by exclusively excluding participants with missing anthropometric data (height/weight) or those meeting any of the following clinical criteria at baseline assessment: (1) self-reported physician-diagnosed hypertension or DM; (2) measured blood pressure ≥140/90 mmHg; (3) current use of antihypertensive or glucose-lowering medications. Participants who were lost to follow-up were excluded in subsequent analyses. Through screening, we ultimately selected a total of 5,342 samples for hypertension, 6,335 for DM, and 6,414 for hypertension-diabetes comorbidity from an initial sample of 16,954 participants.

### Outcomes and exposure

The exposure of interest was BMI. Participants were categorized into four groups according to Chinese guidelines: underweight (BMI < 18.5 kg/m^2^); normal weight (BMI 18.5–23.9 kg/m^2^); overweight (BMI 24.0–27.9 kg/m^2^); and obesity (BMI ≥ 28.0 kg/m^2^) (20). The primary outcome was hypertension-diabetes comorbidity and the secondary outcomes was either hypertension or DM. Hypertension was identified by self-reported physician diagnosis, current use of antihypertensive medications, or average of two blood pressure measurements ≥140/90 mmHg. DM was determined by self-reported physician diagnosis or current use of hypoglycemic medications. Survival time was defined as the period from baseline enrollment to the occurrence of the specified outcomes.

### Follow-up

Our study utilized data from 2008–2009 as the baseline, with follow-up surveys conducted in 2011–2012, 2014, and 2017–2018, targeting participants aged 65 years and older. The follow-up protocol employed two distinct questionnaires: (1) a survivor questionnaire administered to living participants, covering demographic and socioeconomic characteristics, family structure, economic status, self-rated health and quality of life, cognitive function, psychological traits, activities of daily living (ADL), lifestyle factors, caregiving support, and healthcare utilization/costs; and (2) a deceased participant questionnaire completed by immediate family members, which collected detailed retrospective data on the decedent’s pre-mortem health status, quality of life, and medical/caregiving expenditure. This dual-questionnaire design ensured comprehensive longitudinal data capture while accounting for mortality-associated information loss. Detailed follow-up plans can be accessed on the online webpage:

https://opendata.pku.edu.cn/dataset.xhtml?persistentId=doi%3A10.18170%2FDVN%2FWBO7LK

### Statistics analyses

To ensure data quality, we excluded participants with missing values in key variables including BMI, hypertension status, and diabetes status. For candidate covariates (selected based on prior literature), we removed variables with missingness exceeding 20%, while those with ≤20% missing values were considered Missing at Random and were handled using multiple imputation with the predictive mean matching method.

Continuous variables were reported as the mean ± standard deviation (SD) for normally distributed data and median [interquartile range (IQR)] for non-normally distributed data. Statistical differences between groups were assessed using the independent samples t-test for normally distributed variables and the Mann-Whitney U test for non-normally distributed variables. Categorical variables were presented as frequencies (percentages) and compared between groups using either the χ^2^ test or Fisher’s exact test, as appropriate. A two-sided p-value of less than 0.05 was considered statistically significant across all analyses.

Based on BMI categories, we initially employed univariate Cox regression analysis to explore the relationship between candidate variables and all outcomes. We tested the proportional hazards assumption for exposure variable (continuous variable: BMI, categorical variable: BMI groups) using Schoenfeld residuals. Variables with P < 0.05 in the univariate analysis were subsequently included in the multivariate regression model. Additionally, we repeated these analyses with BMI treated as a continuous variable to provide a quantitative assessment and examine the robustness of our findings.

Kaplan-Meier curves were plotted for hypertension, DM, and hypertension-diabetes comorbidity in BMI groups. To address mortality as a competing risk and minimize potential overestimation of outcomes, we implemented a competing risks regression model for analysis (21). The incidence rates of hypertension, DM, and hypertension-diabetes comorbidity at the end of follow-up were also calculated.

To mitigate potential overadjustment bias inherent in data-driven variable selection, we established a Directed Acyclic Graph (DAG) framework incorporating all candidate variables (22). This methodology enabled identification of an optimal adjustment set while preventing overcorrection. Based on this adjustment set, we adopted a multi-model analyses strategy: Model 1 was unadjusted, while Model 2 incorporated age, ethnicity, current exercise status, and total income of year for adjustment.

We employed restricted cubic splines (RCS) to model the association between BMI and hazard ratios (HR). This nonparametric approach effectively captures potential nonlinear relationships while minimizing information loss inherent in categorical classifications. By constraining curve fluctuations through predefined knots (n = 5), RCS ensures robust and interpretable results, thereby providing precise characterization of risk trends across the full BMI spectrum (23). Moreover, subgroup analyses were performed to investigate potential interactions of variables of interest and BMI.

## Results

### Baseline characteristics of CLHLS cohort

**Figure 1:**
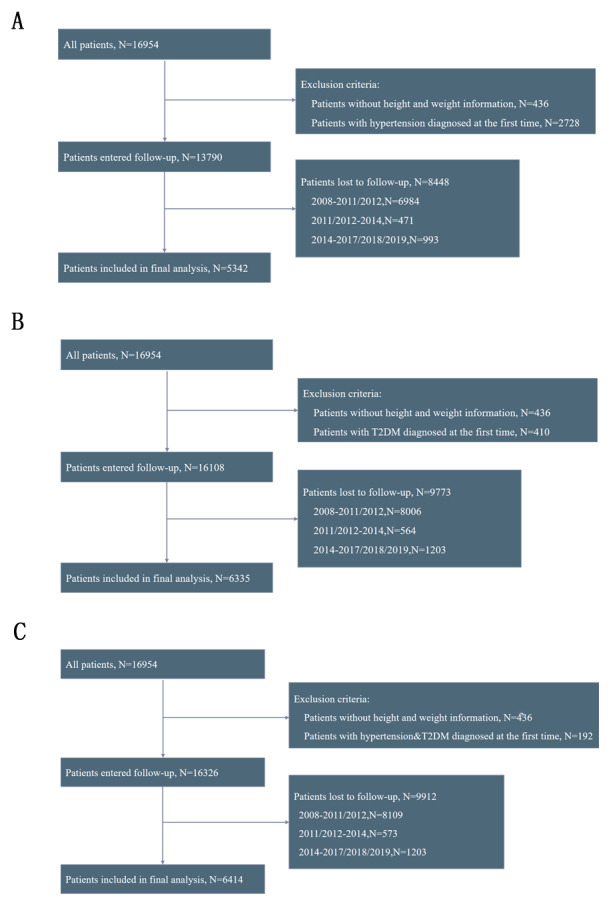
Flowchart of participants screening. **(A)** hypertension; **(B)** diabetes; **(C)** hypertension-diabetes comorbidity. Note: The participants were first enrolled in 2008, with the first follow-up conducted in 2011 and 2012, the second follow-up in 2014, and the third follow-up in 2017, 2018, and 2019.

From an initial sample of 16,954 participants in the CLHLS database, we screened three datasets for analyzing hypertension, DM, and hypertension-diabetes comorbidity, comprising 5,342, 6,335, and 6,414 participants, respectively (Figure 1). In the dataset analyzing hypertension-diabetes comorbidity, the median age was 88.00 years (IQR, 79.50–94.00) for the underweight group, 82.00 years (IQR, 73.00–90.00) for the normal weight group, 78.00 years (IQR, 70.00–87.00) for the overweight group, and 75.00 years (IQR, 68.00–86.00) for the obesity group (P < 0.001). The numbers of male patients were 1,189 (63.5%), 1,786 (50.2%), 395 (49.6%), and 110 (59.1%), respectively (P < 0.001). The numbers of Han ethnicity participants were 1,689 (90.3%), 3,343 (93.9%), 769 (96.5%), and 182 (97.8%), respectively (P < 0.001). At the end of follow-up, the numbers of participants developing hypertension-diabetes comorbidity in the four groups were 283 (15.1%), 719 (20.0%), 235 (29.5%), and 57 (30.6%), respectively (P < 0.001); detailed information is provided in Table 1. Tables S1 and S2 present comprehensive baseline characteristics for the hypertension and DM datasets, respectively.

**Table 1:** Baseline characteristics of participants for the outcome of hypertension-diabetes comorbidity.


VARIABLES	UNDER WEIGHT (n = 1871)	NORMAL WEIGHT (n = 3522)	OVERWEIGHT (n = 774)	OBESITY (n = 180)	P

BMI	17.26[16.02,17.83]	20.81[19.63,22.22]	25.26[24.46,26.26]	29.81[28.82,31.59]	<0.001

Age, years	88.00[79.50,94.00]	82.00[73.00,90.00]	78.00[70.00,87.00]	75.00[68.00,86.00]	<0.001

Male	1189(63.5)	1786(50.2)	395(49.6)	110(59.1)	<0.001

Ethnic					<0.001

Han	1689(90.3)	3343(93.9)	769(96.5)	182(97.8)	

Hui	6(0.3)	11(0.3)	5(0.6)	2(1.1)	

Zhuang	142(7.6)	109(3.1)	13(1.6)	1(0.5)	

Yao	11(0.6)	18(0.5)	1(0.1)	0(0.0)	

Korea	10(0.5)	16(0.4)	2(0.3)	0(0.0)	

Mongolia	0(0.0)	1(0.0)	0(0.0)	0(0.0)	

Others	13(0.7)	62(1.7)	7(0.9)	1(0.5)	

Current smoking status					<0.001

Yes	320(17.1)	787(22.1)	170(21.3)	27(14.5)	

No	1551(82.9)	2773(77.9)	627(78.7)	159(85.5)	

Current drinking status					<0.001

Yes	283(15.1)	790(22.2)	181(22.7)	36(19.4)	

No	1588(84.9)	2770(77.8)	616(77.3)	150(80.6)	

Current exercise status					<0.001

Yes	468(25.0)	1097(30.8)	317(39.8)	74(39.8)	

No	1403(75.0)	2463(69.2)	480(60.2)	112(60.2)	

Total income of year, CNY	10000.00[3500.00,23000.00]	10000.00[4000.00,24250.00]	10000.00[5000.00,25000.00]	10000.00[4000.00,25000.00]	0.102

Current marital status					<0.001

Currently married and living with spouse	527(28.2)	1496(42.0)	413(51.8)	98(52.7)	

Separated	44(2.4)	90(2.5)	12(1.5)	2(1.1)	

Divorced	6(0.3)	4(0.1)	7(0.9)	3(1.6)	

Widowed	1278(68.3)	1939(54.5)	359(45.0)	82(44.1)	

Never married	16(0.9)	31(0.9)	6(0.8)	1(0.5)	

Sleep quality					<0.001

Very good	209(11.2)	530(14.9)	125(15.7)	36(19.4)	

Good	925(49.4)	1926(54.1)	442(55.5)	104(55.9)	

So so	504(26.9)	792(22.2)	152(19.1)	30(16.1)	

Bad	215(11.5)	284(8.0)	69(8.7)	15(8.1)	

Very bad	17(0.9)	22(0.6)	7(0.9)	1(0.5)	

Not able to answer	1(0.1)	6(0.2)	2(0.3)	0(0.0)	

Sleep duration, hours	8.00[6.00,9.00]	8.00[6.00,10.00]	8.00[7.00,10.00]	8.00[7.00,10.00]	0.084

Hypertension and DM	283(15.1)	719(20.2)	235(29.5)	57(30.6)	<0.001


BMI: Body mass index, CNY: Chinese Yuan. BMI category: underweight (BMI < 18.5 kg/m^2^), normal weight (BMI 18.5–23.9 kg/m^2^), overweight (BMI 24.0–27.9 kg/m^2^), and obesity (BMI ≥ 28.0 kg/m^2^).

Additionally, during median follow-up periods of 71 months (IQR: 41–117), 75 months (IQR: 71–121), and 74 months (IQR: 71–120), the incidence rates of hypertension, DM, and hypertension-diabetes comorbidity were found to be 6.09/1000 person-months, 2.36/1000 person-months, and 2.22/1000 person-months, respectively.

### Survival analysis and competing risks model

We tested the proportional hazards assumption for the exposure variable using Schoenfeld residuals. The results indicated that BMI, both as a continuous variable and a categorical variable, satisfied the proportional hazards assumption (Figures S1-S2 for hypertension-diabetes comorbidity, Figures S3-S4 for hypertension, and Figures S5-S6 for diabetes comorbidity).

**Figure 2:**
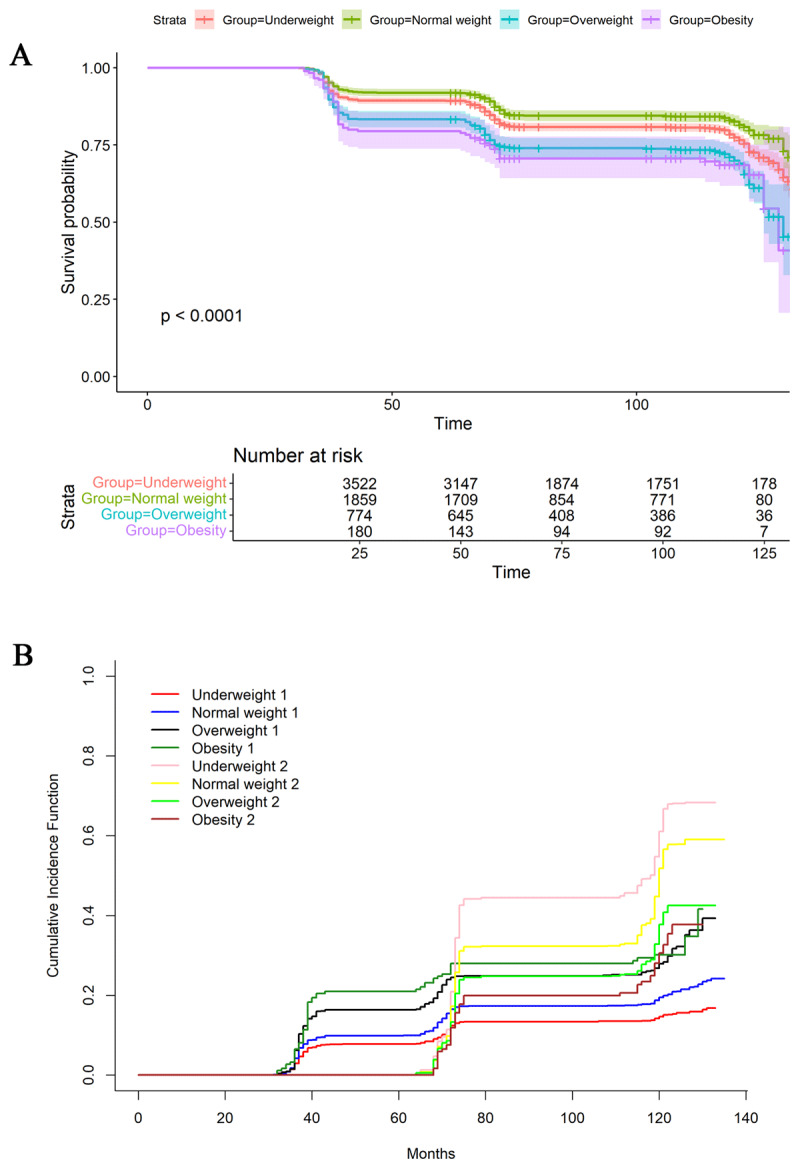
Survival analysis **(A)** and competing risks analysis regarding death **(B)** were performed for hypertension-diabetes comorbidity. Participants were categorized into four groups based on BMI: underweight (BMI < 18.5 kg/m^2^), normal weight (BMI 18.5–23.9 kg/m^2^), overweight (BMI 24.0–27.9 kg/m^2^), and obesity (BMI ≥ 28.0 kg/m^2^). **(A)** “1” indicates the occurrence of hypertension-diabetes comorbidity and “2” indicates the occurrence of death.

Survival analysis revealed varying risks of the hypertension-diabetes comorbidity in different BMI groups. The obesity group exhibited the highest risk, and the underweight group had the lowest risk (log-rank P < 0.001; Figure 2A).

Given the advanced age and high mortality risk of the study participants, we further considered death as a competing risk (Figure 2B). Even after accounting for this, the higher BMI had higher risk for all outcomes (P < 0.001).

The survival analysis and competing risks analysis results for hypertension (Figure S7A and S7B) and DM (Figure S8A and S8B) were consistent with those for hypertension-diabetes comorbidity.

### Cox regression analysis based on a data-driven strategy

In the analysis of hypertension-diabetes comorbidity, the univariate analysis identified several significant variables: BMI (categorical), age, ethnicity, current exercise status, current marital status, total income of year, and sleep duration. After multivariate adjustment, the significant variables that remained were age, BMI, ethnicity, current marital status, total income of year, and sleep duration. The corresponding HRs were as follows: age, 0.985 (95% CI, 0.979–0.992); BMI categories, with underweight at 0.837 (95% CI, 0.728–0.692), overweight at 1.397 (95% CI, 1.204–1.621), and obesity at 1.438 (95% CI, 1.096–1.886) versus normal weight; ethnicity, with non-Han at 0.334 (95% CI, 0.235–0.475) versus Han; current marital status, with widowed at 2.828 (95% CI, 1.169–6.839) and separated at 2.367 (95% CI, 0.906–6.187) versus never married; total income or year at 1.001 (95% CI, 1.001–1.001); and sleep duration at 1.014 (95% CI, 1.005–1.022). When BMI was reanalyzed as a continuous variable, the significance analysis aligned with the aforementioned results, indicating the robustness of the findings (Table 2).

**Table 2:** Univariate and multivariate Cox regression of BMI and hypertension-diabetes comorbidity.


VARIABLES	UNIVARIATE, HR (95%CI)	P	MULTIVARIATE^a^, HR (95%CI)	P^a^	MULTIVARIATE^b^, HR (95%CI)	P^b^

BMI group						

Normal weight	1.000(Reference)		1.000(Reference)			

Underweight	0.747(0.651,0.857)	<0.001	0.837(0.728,0.962)	0.013		

Overweight	1.517(1.309,1.758)	<0.001	1.397(1.204,1.621)	<0.001		

Obesity	1.620(1.237,2.121)	<0.001	1.438(1.096,1.886)	0.009		

BMI	1.062(1.048,1.076)	<0.001			1.043(1.029,1.059)	<0.001

Age, years	0.980(0.975,0.986)	<0.001	0.985(0.979,0.992)	<0.001	0.985(0.979,0.991)	<0.001

Sex						

Female	1.000(Reference)					

Male	0.950(0.851,1.059)	0.353				

Ethnic						

Han	1.000(Reference)		1.000(Reference)		1.000(Reference)	

Non-han	0.301(0.212,0.428)	<0.001	0.334(0.235,0.475)	<0.001	0.333(0.234,0.474)	<0.001

Current smoking status						

No	1.000(Reference)					

Yes	1.072(0.939,1.224)	0.304				

Current drinking status						

No	1.000(Reference)					

Yes	1.100(0.964,1.255)	0.158				

Current exercise status						

No	1.000(Reference)		1.000(Reference)		1.000(Reference)	

Yes	1.227(1.094,1.375)	<0.001	1.094(0.975,1.228)	0.126	1.091(0.972,1.225)	0.141

Current marital status						

Never married	1.000(Reference)		1.000(Reference)		1.000(Reference)	

Widowed	2.067(0.857,4.985)	0.106	2.828(1.169,6.839)	0.021	2.789(1.153,6.750)	0.023

Currently married and living with spouse	2.855(1.184,6.883)	0.019	2.367(0.906,6.187)	0.079	2.326(0.890,6.081)	0.085

Divorced	2.452(0.658,9.133)	0.181	2.026(0.543,7.555)	0.293	2.049(0.550,7.636)	0.285

Separated	1.930(0.741,5.027)	0.178	2.561(1.057,6.209)	0.037	2.534(1.045,6.146)	0.040

Total income of year, CNY	1.001(1.001,1.001)	0.027	1.001(1.001,1.001)	0.030	1.001(1.001,1.001)	0.037

Sleep duration, hours	1.011(1.002,1.020)	0.016	1.014(1.005,1.022)	0.002	1.014(1.005,1.022)	0.002

Sleep quality						

Very good	1.000(Reference)					

Good	0.917(0.781,1.077)	0.291				

Bad	0.952(0.759,1.196)	0.675				

So so	0.938(0.783,1.124)	0.488				

Very bad	1.277(0.728,2.240)	0.393				

Not able to answer	0.546(0.077,3.898)	0.546				


HR: Hazard ratio, CI: Confidence interval, BMI: Body mass index, CNY: Chinese Yuan. BMI category: underweight (BMI < 18.5 kg/m^2^), normal weight (BMI 18.5–23.9 kg/m^2^), overweight (BMI 24.0–27.9 kg/m^2^), and obesity (BMI ≥ 28.0 kg/m^2^). ^a^: When conducting multivariate Cox regression analysis, BMI is included in the model as a categorical variable. ^b^: When conducting multivariate Cox regression analysis, BMI is included in the model as a continuous variable.

Furthermore, in the dataset with hypertension as the outcome, the significant variables that remained after adjustment were BMI (categorical), age, and ethnicity. Within the BMI categories, higher BMI groups exhibited a greater risk of developing hypertension. Compared to the Han ethnicity, non-Han individuals had a significantly lower risk of hypertension. When BMI was included in the model as a continuous variable, the significance analysis results were consistent with those obtained when it was treated as a categorical variable (Table S3). In the dataset with DM as the outcome, the significance analysis results were consistent with those for hypertension-diabetes comorbidity (Table S4).

### A multi-model adjustment strategy based on DAG

**Figure 3:**
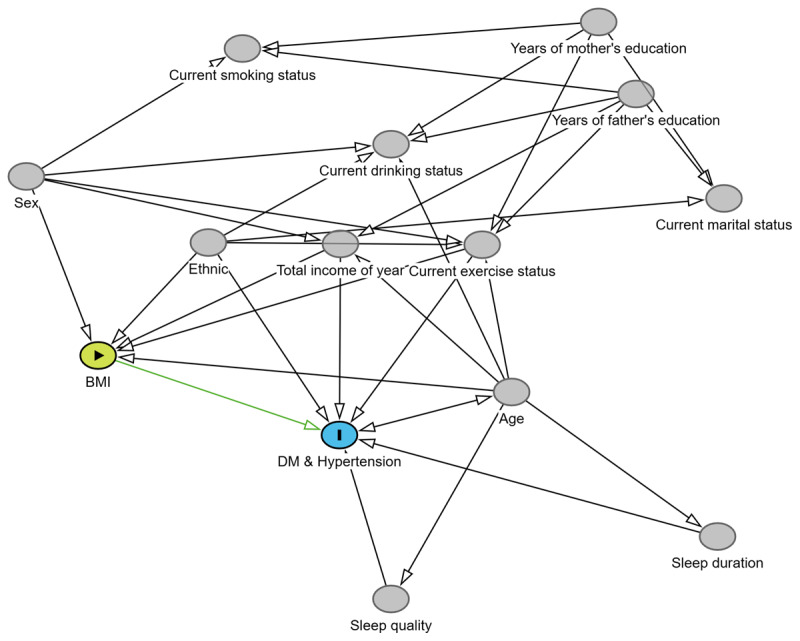
Directed Acyclic Graph (DAG) with Body Mass Index (BMI) as the exposure and hypertension-diabetes comorbidity as the outcome.

Considering that data-driven strategies may lead to over-adjustment, we employed DAG analysis (Figure 3) to identify the minimal adjustment set: age, ethnicity, current exercise status, and total income of year. When the outcome was hypertension-diabetes comorbidity and BMI was treated as a categorical variable (BMI ≥ 24.0 versus BMI < 24.0), the HR of Model 1 (unadjusted) was 1.682 (95% CI, 1.476–1.917). After using the minimal adjustment set, the HR was 1.486 (95% CI, 1.301–1.698). When BMI was treated as a continuous variable, the significance analysis aligned with the aforementioned results (Table 3). When the outcomes were hypertension or DM, separate DAG (Figure S9 and Figure S10) were constructed for each condition to perform multi-model analyses. The results were consistent with those observed for hypertension-diabetes comorbidity (Table 3).

**Table 3:** Multi-model adjustment based on Directed Acyclic Graph (DAG).


DISEASES	GROUP	MODEL 1 HR (95%CI)	P	MODEL 2 HR (95%CI)	P

Hypertension & DM	BMI<24.0	1.000(Reference)		1.000(Reference)	

	BMI≥24.0	1.682(1.476~1.917)	<0.001	1.486(1.301~1.698)	<0.001

	BMI*	1.062(1.048~1.076)	<0.001	1.046(1.031~1.061)	<0.001

Hypertension	BMI<24.0	1.000(Reference)		1.000(Reference)	

	BMI≥24.0	1.499(1.351~1.662)	<0.001	1.398(1.258~1.552)	<0.001

	BMI*	1.052(1.041~1.062)	<0.001	1.042(1.031~1.053)	<0.001

DM	BMI<24.0	1.000(Reference)		1.000(Reference)	

	BMI≥24.0	1.639(1.440~1.865)	<0.001	1.451(1.271~1.656)	<0.001

	BMI*	1.060(1.046~1.074)	<0.001	1.044(1.029~1.059)	<0.001


HR: Hazard Ratio, CI: Confidence Interval, BMI: Body mass index, DM: diabetes mellitus. Model1: Crude. Model2: Adjust: age, ethnic, current exercise status, total income of year.

### RCS and subgroup analyses

**Figure 4:**
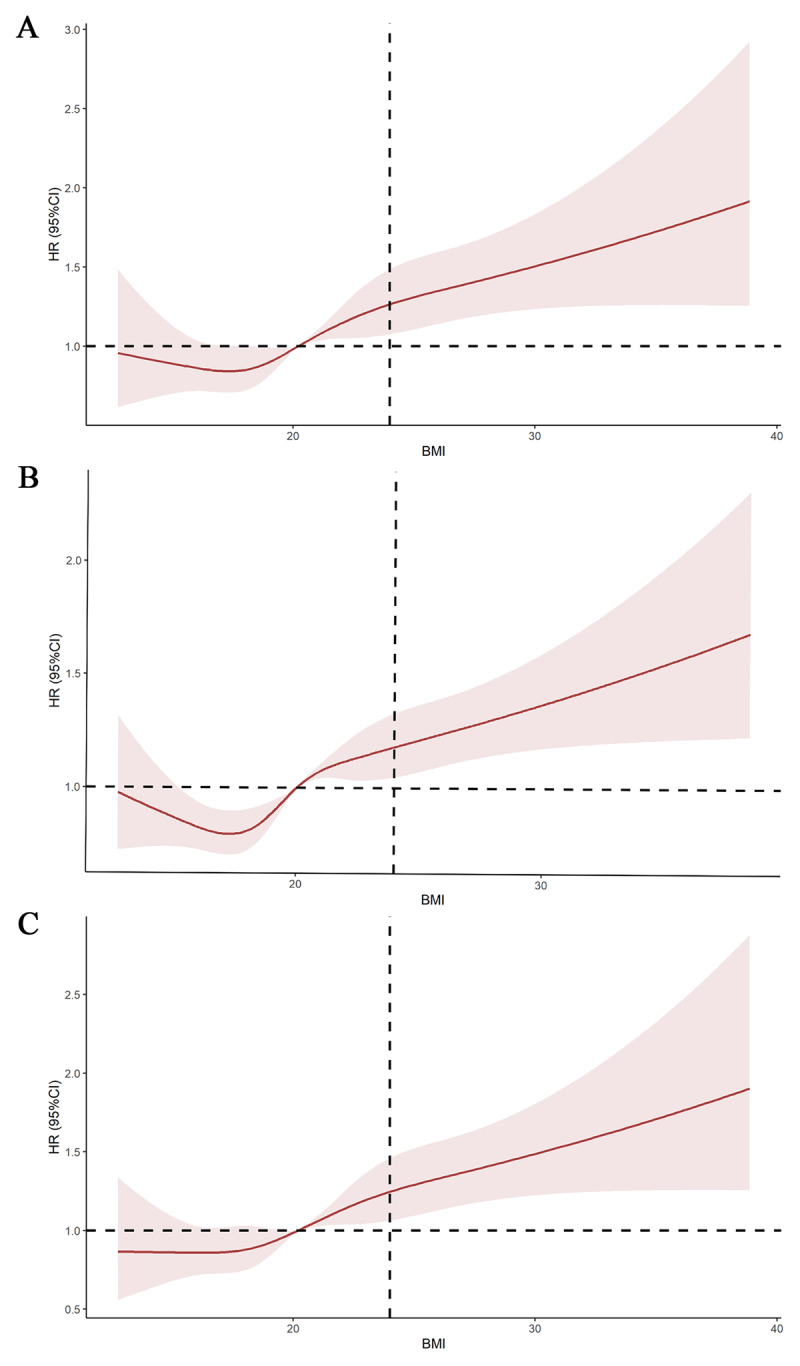
Restricted cubic spline (RCS) fitting the relationship between body mass index (BMI) as a continuous variable and related outcomes. A represents hypertension, B represents diabetes mellitus, and C represents hypertension-diabetes comorbidity.

**Figure 5:**
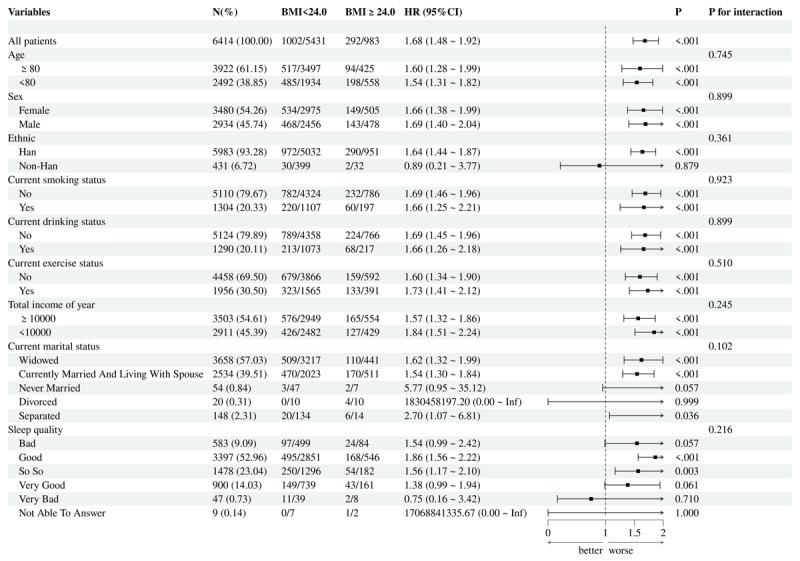
Subgroup analysis for hypertension-diabetes comorbidity with body mass index (BMI) as the exposure.

RCS analysis indicated a linear positive correlation between BMI and the risk of all outcomes (Figure 4). This finding further indicates that there is a cumulative dose effect for BMI. Additionally, this study stratified the study population into multiple subgroups based on age, gender, ethnicity, current smoking status, current drinking status, current exercise status, total income of year, sleep quality, and current marital status. The subgroup analysis results for hypertension-diabetes comorbidity (Figure 5), hypertension (Figure S11), and DM (Figure S12) showed that the P-values for interaction were all greater than 0.05 across all variables, indicating no interactive effects among these variables. Overall, BMI ≥ 24.0 was identified as a reliable risk factor for all three outcomes.

## Discussion

To the best of our knowledge, this is the first prospective cohort study conducted in China that explores the association between BMI and hypertension, DM, as well as hypertension-diabetes comorbidity specifically within the elderly population. By examining BMI both as a categorical and continuous variable on outcomes, this study demonstrated that BMI increased the risk of hypertension-diabetes comorbidity, hypertension, and DM. Additionally, Han participants (versus non-Han) have a notably greater risk of developing hypertension-diabetes comorbidity, hypertension, and DM. Widowed or separated individuals have an elevated risk of developing hypertension-diabetes comorbidity and DM compared to unmarried individuals. This study suggests that greater attention should be given to obesity among elderly Han Chinese individuals, particularly those who are widowed, as they face a significantly higher risk of chronic diseases.

### The relationship between BMI and hypertension-diabetes comorbidity

In this study, we found that higher BMI significantly increases the risk of hypertension-diabetes comorbidity, showing a higher HR compared to when hypertension or DM is considered as individual outcomes. No large-scale prospective cohort studies have previously investigated this issue, but some studies may provide support for our findings. Wu et al. (19) conducted a cross-sectional study among participants aged 18 and above in Hubei Province, China, showing that higher BMI increases the risk of hypertension-diabetes comorbidity. Li et al. (18) performed a retrospective cohort study among participants aged 60 and older in Henan Province, China, indicating that higher BMI increases the risk of hypertension-diabetes comorbidity. Additionally, a cross-sectional study in England explored the relationship between generalized and abdominal obesity and hypertension-diabetes comorbidity in individuals aged 35 and older, revealing that both indicators increase the risk of hypertension-diabetes comorbidity (24).

When DM and hypertension coexist, they can mutually influence each other, further exacerbating cardiac function (25). Diabetes can lead to impaired endothelial function and increase the risk of arteriosclerosis, thereby aggravating hypertension. Conversely, hypertension can worsen insulin resistance, further exacerbating DM (26,27). DM and hypertension often exhibit familial aggregation and are associated with unhealthy lifestyles (28,29). Since obesity is a significant risk factor for both DM and hypertension, controlling BMI to reduce the risk of hypertension-diabetes comorbidity is feasible.

### The relationship between BMI and hypertension

This study demonstrates that a higher BMI is associated with an increased risk of hypertension. The risk factors for hypertension are multifaceted, with previous research primarily encompassing genetic, environmental, and lifestyle aspects (30). Overweight and obesity, particularly central obesity, constitute significant risk factors for the development of hypertension (31,32,33,34). Specifically, as BMI increases, blood pressure levels tend to rise accordingly. One study revealed that for every one standard deviation increase in BMI, the odds ratio for hypertension increases by 1.42 (95% CI, 1.37–1.48) (35). Currently, it is believed that mechanisms such as increased blood volume load, insulin resistance, changes in peripheral resistance vessels, and activation of the renin-angiotensin system may underlie the influence of BMI on hypertension (36,37).

### The relationship between BMI and DM

The risk factors for DM are multifaceted, including genetic factors, obesity, unhealthy lifestyles, and aging (38,39,40,41). Our study demonstrates that higher BMI increases the risk of DM. Similarly, Tang et al. (42) found that high BMI was associated with a high risk of developing DM in the Chinese aged population. Obesity stands as one of the crucial risk factors for the development of DM. Both the duration and degree of obesity are closely related to the onset of DM (43,44). Central obesity, also known as abdominal obesity, poses the highest risk for developing DM (45). Obesity leads to reduced insulin sensitivity and weakened regulation of blood glucose, thereby increasing the risk of DM (46,47).

### Other Valuable Findings

Compared to unmarried participants, being widowed or separated after marriage results in an increased risk of both DM and hypertension-diabetes comorbidity. These findings are quite robust in our study. In the Australian Longitudinal Study on Women’s Health (ALSWH), it was found that women aged 40–45 years who were separated or widowed had an increased risk of developing DM (48). A study conducted in the United States indicated that being widowed increases the risk of hypertension (49). This study further confirms it and broadens the population under investigation.

Additionally, this study revealed that non-Han ethnic has significantly lower risk of developing hypertension, DM, and hypertension-diabetes comorbidity compared to the Han ethnic. This conclusion is similar to the findings of Wang et al. (50), but our study further expands the ethnic diversity. These observed disparities likely stem from multifaceted socioeconomic and lifestyle differences. Notably, epidemiological surveys indicate that Tibetan populations, as one representative non-Han group, tend to maintain more physically active lifestyles rooted in traditional pastoral practices, along with distinct dietary patterns characterized by habitual consumption of butter tea (a mixture of tea leaves, yak butter, and salt) which differs nutritionally from typical Han diets (50). These ethnocultural variations in daily energy expenditure and nutritional intake patterns may contribute to the differential cardiometabolic risk profiles observed across ethnicities. Disparities in healthcare resource distribution across China, along with the elevated metabolic risks potentially associated with the Han ethnic group’s higher urbanization rate (51,52).

As the largest ethnic group in China, the Han population has increased demands for disease prevention. Our study contributes to the development of relevant guidelines to address these needs.

### Limitations

Firstly, although standard procedures were employed for the physical examinations, measurement bias may exist due to the high prevalence of osteoporosis and kyphosis among the elderly. Secondly, these results are based solely on a single BMI measurement at baseline, and the potential impact of social or healthcare changes over time was not fully controlled. Thirdly, selection bias resulting from the exclusion of lost-to-follow-up individuals may have influenced the study results. Finally, we did not systematically evaluate potential confounding effects from other chronic comorbidities on the BMI-comorbidity association.

Despite these limitations, compared to previous studies, this study provides more robust evidence. Future research should focus on optimizing measurement methods and data collection, considering the influence of time factors and social changes, reducing self-report bias, controlling for selection bias.

## Conclusions

This study found that a higher BMI is significantly associated with an increased risk of hypertension-diabetes comorbidity, hypertension and DM, with a linear trend observed between BMI (range in BMI > 24.0) and the risk of these outcomes. Furthermore, compared to other ethnic, the Han ethnic exhibits a higher risk of developing hypertension and hypertension-diabetes comorbidity. Marital statuses of being widowed and separated were found to elevate the risk of DM and hypertension-diabetes comorbidity. These findings highlight that the disease burden attributable to BMI is more severe in the Han Chinese population, underscoring the critical need for targeted prevention policies.

## Data Accessibility Statement

The data supporting the findings of this article is available on the online webpage: https://opendata.pku.edu.cn/dataset.xhtml?persistentId=doi%3A10.18170%2FDVN%2FWBO7LK.

## Additional File

The additional file for this article can be found as follows:

10.5334/gh.1487.s1 Supplementary Files. Supplementary Figures S1 to S12 and Tables S1 to S4.

## References

[1] Lu J, Lu Y, Wang X, et al. Prevalence, awareness, treatment, and control of hypertension in China: Data from 1.7 million adults in a population-based screening study (China PEACE Million Persons Project). Lancet. 2017;390:2549–2558. DOI: 10.1016/S0140-6736(17)32478-9 29102084

[2] Mohammadian Khonsari N, Shahrestanaki E, Ejtahed HS, et al. Long-term trends in hypertension prevalence, awareness, treatment, and control rate in the Middle East and North Africa: A systematic review and meta-analysis of 178 population-based studies. Curr Hypertens Rep. 2021;23:41. DOI: 10.1007/s11906-021-01159-0 34625888

[3] Fan Z, Yang C, Zhang J, et al. Trends and influence factors in the prevalence, awareness, treatment, and control of hypertension among US adults from 1999 to 2018. PLoS One. 2023;18:e0292159. DOI: 10.1371/journal.pone.0292159 37768964 PMC10538741

[4] Aviles-Santa ML, Monroig-Rivera A, Soto-Soto A, Lindberg NM. Current state of diabetes mellitus prevalence, awareness, treatment, and control in Latin America: Challenges and innovative solutions to improve health outcomes across the continent. Curr Diab Rep. 2020;20:62. DOI: 10.1007/s11892-020-01341-9 33037442 PMC7546937

[5] Wang L, Peng W, Zhao Z, et al. Prevalence and treatment of diabetes in China, 2013–2018. JAMA. 2021;326:2498–2506. DOI: 10.1001/jama.2021.22208 34962526 PMC8715349

[6] Elmaleh-Sachs A, Schwartz JL, Bramante CT, et al. Obesity management in adults: A review. JAMA. 2023;330:2000–2015. DOI: 10.1001/jama.2023.19897 38015216 PMC11325826

[7] Laouali N, Shah S, MacDonald CJ, et al. BMI in the associations of plant-based diets with type 2 diabetes and hypertension risks in women: The E3N prospective cohort study. J Nutr. 2021;151:2731–2740. DOI: 10.1093/jn/nxab158 34236437

[8] Ouyang X, Lou Q, Gu L, et al. Anthropometric parameters and their associations with cardio-metabolic risk in Chinese working population. Diabetol Metab Syndr. 2015;7:37. DOI: 10.1186/s13098-015-0032-5 25960779 PMC4424518

[9] Benabdelkamel H, Masood A, Okla M, Al-Naami MY, Alfadda AA. A proteomics-based approach reveals differential regulation of urine proteins between metabolically healthy and unhealthy obese patients. Int J Mol Sci. 2019;20. DOI: 10.3390/ijms20194905 PMC6801506 31623319

[10] Du Z, Zhu W, Zhao Y, et al. The epidemic of stroke mortality attributed to high body mass index in mainland China: Current trends and future prediction. Front Public Health. 2022;10:1021646. DOI: 10.3389/fpubh.2022.1021646 36353279 PMC9639780

[11] Lin H, Xiao N, Lin S, Liu M, Liu GG. Associations of hypertension, diabetes and heart disease risk with body mass index in older Chinese adults: A population-based cohort study. BMJ Open. 2024;14:e083443. DOI: 10.1136/bmjopen-2023-083443 PMC11268030 38986550

[12] Lip S, Jeemon P, McCallum L, et al. Contrasting mortality risks among subgroups of treated hypertensive patients developing new-onset diabetes. Eur Heart J. 2016;37:968–974. DOI: 10.1093/eurheartj/ehv557 26508167 PMC5841224

[13] Bruno RM, Taddei S. New-onset diabetes in hypertensive patients and mortality: Timing is everything. Eur Heart J. 2016;37:975–977. DOI: 10.1093/eurheartj/ehv594 26553544

[14] Gupta AK, Dahlof B, Dobson J, et al. Determinants of new-onset diabetes among 19,257 hypertensive patients randomized in the Anglo-Scandinavian Cardiac Outcomes Trial--Blood Pressure Lowering Arm and the relative influence of antihypertensive medication. Diabetes Care. 2008;31:982–988. DOI: 10.2337/dc07-1768 18235048

[15] Ferrannini E, Buzzigoli G, Bonadonna R, et al. Insulin resistance in essential hypertension. N Engl J Med. 1987;317:350–357. DOI: 10.1056/NEJM198708063170605 3299096

[16] Kahn BB. Type 2 diabetes: When insulin secretion fails to compensate for insulin resistance. Cell. 1998;92:593–596. DOI: 10.1016/S0092-8674(00)81125-3 9506512

[17] Boarescu PM, Boarescu I, Pop RM, et al. Evaluation of oxidative stress biomarkers, pro-inflammatory cytokines, and histological changes in experimental hypertension, dyslipidemia, and type 1 diabetes mellitus. Int J Mol Sci. 2022;23. DOI: 10.3390/ijms23031438 PMC8835716 35163364

[18] Li H, Shi Z, Chen X, et al. Relationship between obesity indicators and hypertension-diabetes comorbidity in an elderly population: A retrospective cohort study. BMC Geriatr. 2023;23:789. DOI: 10.1186/s12877-023-04510-z 38036950 PMC10691080

[19] Wu W, Wu Y, Yang J, et al. Relationship between obesity indicators and hypertension-diabetes comorbidity among adults: A population study from Central China. BMJ Open. 2022;12:e052674. DOI: 10.1136/bmjopen-2021-052674 PMC9305822 35858720

[20] Chen C, Lu FC, Department of Disease Control Ministry of Health PRC. The guidelines for prevention and control of overweight and obesity in Chinese adults. Biomed Environ Sci. 2004;17Suppl:1–36. 15807475

[21] Hageman SHJ, Dorresteijn JAN, Pennells L, et al. The relevance of competing risk adjustment in cardiovascular risk prediction models for clinical practice. Eur J Prev Cardiol. 2023;30:1741–1747. DOI: 10.1093/eurjpc/zwad202 37338108

[22] Lipsky AM, Greenland S. Causal directed acyclic graphs. JAMA. 2022;327:1083–1084. DOI: 10.1001/jama.2022.1816 35226050

[23] Desquilbet L, Mariotti F. Dose-response analyses using restricted cubic spline functions in public health research. Stat Med. 2010;29:1037–1057. DOI: 10.1002/sim.3841 20087875

[24] Hirani V, Zaninotto P, Primatesta P. Generalised and abdominal obesity and risk of diabetes, hypertension and hypertension-diabetes co-morbidity in England. Public Health Nutr. 2008;11:521–527. DOI: 10.1017/S1368980007000845 17767799

[25] Russo C, Jin Z, Homma S, et al. Effect of diabetes and hypertension on left ventricular diastolic function in a high-risk population without evidence of heart disease. Eur J Heart Fail. 2010;12:454–461. DOI: 10.1093/eurjhf/hfq022 20211851 PMC2857987

[26] Izzo R, de Simone G, Trimarco V, et al. Hypertensive target organ damage predicts incident diabetes mellitus. Eur Heart J. 2013;34:3419–3426. DOI: 10.1093/eurheartj/eht281 23882068 PMC3836008

[27] Zuloaga KL, Johnson LA, Roese NE, et al. High fat diet-induced diabetes in mice exacerbates cognitive deficit due to chronic hypoperfusion. J Cereb Blood Flow Metab. 2016;36:1257–1270. DOI: 10.1177/0271678X15616400 26661233 PMC4929700

[28] Shen XM, Huang YQ, Zhang XY, et al. Association between dietary patterns and prediabetes risk in a middle-aged Chinese population. Nutr J. 2020;19:77. DOI: 10.1186/s12937-020-00593-1 32731880 PMC7393887

[29] He Y, Li Y, Yang X, et al. The dietary transition and its association with cardiometabolic mortality among Chinese adults, 1982–2012: a cross-sectional population-based study. Lancet Diabetes Endocrinol. 2019;7:540–548. DOI: 10.1016/S2213-8587(19)30152-4 31085143 PMC7269053

[30] Kornitzer M, Dramaix M, De Backer G. Epidemiology of risk factors for hypertension: Implications for prevention and therapy. Drugs. 1999;57:695–712. DOI: 10.2165/00003495-199957050-00003 10353295

[31] Gelber RP, Gaziano JM, Manson JE, Buring JE, Sesso HD. A prospective study of body mass index and the risk of developing hypertension in men. Am J Hypertens. 2007;20:370–377. DOI: 10.1016/j.amjhyper.2006.10.011 17386342 PMC1920107

[32] Patel SA, Ali MK, Alam D, et al. Obesity and its relation with diabetes and hypertension: A cross-sectional study across 4 geographical regions. Glob Heart. 2016;11:71–79.e74. DOI: 10.1016/j.gheart.2016.01.003 27102024 PMC4843822

[33] Khan SS, Ning H, Wilkins JT, et al. Association of body mass index with lifetime risk of cardiovascular disease and compression of morbidity. JAMA Cardiol. 2018;3:280–287. DOI: 10.1001/jamacardio.2018.0022 29490333 PMC5875319

[34] Shihab HM, Meoni LA, Chu AY, et al. Body mass index and risk of incident hypertension over the life course: The Johns Hopkins Precursors Study. Circulation. 2012;126:2983–2989. DOI: 10.1161/CIRCULATIONAHA.112.117333 23151344 PMC3743236

[35] van Oort S, Beulens JWJ, van Ballegooijen AJ, Grobbee DE, Larsson SC. Association of cardiovascular risk factors and lifestyle behaviors with hypertension: A mendelian randomization study. Hypertension. 2020;76:1971–1979. DOI: 10.1161/HYPERTENSIONAHA.120.15761 33131310

[36] Seravalle G, Grassi G. Obesity and hypertension. Pharmacol Res. 2017;122:1–7. DOI: 10.1016/j.phrs.2017.05.013 28532816

[37] Hall JE, Mouton AJ, da Silva AA, et al. Obesity, kidney dysfunction, and inflammation: Interactions in hypertension. Cardiovasc Res. 2021;117:1859–1876. DOI: 10.1093/cvr/cvaa336 33258945 PMC8262632

[38] Chandrasekaran P, Weiskirchen R. The role of obesity in type 2 diabetes mellitus-an overview. Int J Mol Sci. 2024;25. DOI: 10.3390/ijms25031882 PMC10855901 38339160

[39] Caleyachetty R, Barber TM, Mohammed NI, et al. Ethnicity-specific BMI cutoffs for obesity based on type 2 diabetes risk in England: A population-based cohort study. Lancet Diabetes Endocrinol. 2021;9:419–426. DOI: 10.1016/S2213-8587(21)00088-7 33989535 PMC8208895

[40] Qin J, Li Y, Cai Z, et al. A metagenome-wide association study of gut microbiota in type 2 diabetes. Nature. 2012;490:55–60. DOI: 10.1038/nature11450 23023125

[41] Ling C, Bacos K, Ronn T. Epigenetics of type 2 diabetes mellitus and weight change – a tool for precision medicine? Nat Rev Endocrinol. 2022;18:433–448. DOI: 10.1038/s41574-022-00671-w 35513492

[42] Tang ML, Zhou YQ, Song AQ, et al. The relationship between body mass index and incident diabetes mellitus in Chinese aged population: A cohort study. J Diabetes Res. 2021;2021:5581349. DOI: 10.1155/2021/5581349 34485532 PMC8410436

[43] Hu Y, Bhupathiraju SN, de Koning L, Hu FB. Duration of obesity and overweight and risk of type 2 diabetes among US women. Obesity (Silver Spring). 2014;22:2267–2273. DOI: 10.1002/oby.20851 25131512 PMC4180760

[44] Bays HE, Chapman RH, Grandy S. The relationship of body mass index to diabetes mellitus, hypertension and dyslipidaemia: Comparison of data from two national surveys. Int J Clin Pract. 2007;61:737–747. DOI: 10.1111/j.1742-1241.2007.01336.x 17493087 PMC1890993

[45] Cao C, Hu H, Zheng X, et al. Association between central obesity and incident diabetes mellitus among Japanese: A retrospective cohort study using propensity score matching. Sci Rep. 2022;12:13445. DOI: 10.1038/s41598-022-17837-1 35927472 PMC9352654

[46] Ahmed B, Sultana R, Greene MW. Adipose tissue and insulin resistance in obese. Biomed Pharmacother. 2021;137:111315. DOI: 10.1016/j.biopha.2021.111315 33561645

[47] Sharma AM, Chetty VT. Obesity, hypertension and insulin resistance. Acta Diabetol. 2005;42 Suppl 1:S3–8. DOI: 10.1007/s00592-005-0175-1 15868117

[48] Xu X, Mishra GD, Dobson AJ, Jones M. Progression of diabetes, heart disease, and stroke multimorbidity in middle-aged women: A 20-year cohort study. PLoS Med. 2018;15:e1002516. DOI: 10.1371/journal.pmed.1002516 29534066 PMC5849280

[49] Pantell MS, Prather AA, Downing JM, Gordon NP, Adler NE. Association of social and behavioral risk factors with earlier onset of adult hypertension and diabetes. JAMA Netw Open. 2019;2:e193933. DOI: 10.1001/jamanetworkopen.2019.3933 31099868 PMC6537925

[50] Wang L, Gao P, Zhang M, et al. Prevalence and ethnic pattern of diabetes and prediabetes in China in 2013. JAMA. 2017;317:2515–2523. DOI: 10.1001/jama.2017.7596 28655017 PMC5815077

[51] Chen S, Si Y, Hanewald K, et al. Disease burden of ageing, sex and regional disparities and health resources allocation: A longitudinal analysis of 31 provinces in Mainland China. BMJ Open. 2022;12:e064641. DOI: 10.1136/bmjopen-2022-064641 PMC9670959 36385040

[52] Luo F, Huang Y, Jiang L, Fan Q, Gou Z. Ethnic disparities and temporal trends in health resource allocation: A retrospective decadal analysis in Sichuan, a multi-ethnic Province of Southwest China (2009–2019). BMC Health Serv Res. 2024;24:541. DOI: 10.1186/s12913-024-11036-6 38678273 PMC11056051

