# Supplementary material for: Body Mass Index and the Risk of Hypertension-Diabetes Comorbidity in Elderly Population: A Prospective Cohort in China
Source: Glob Heart. 2025 Oct 16;20(1):97. doi: 10.5334/gh.1487 (PMC12533419; doi:10.5334/gh.1487)
Supplement: Supplementary Files. — Supplementary Figures S1 to S12 and Tables S1 to S4. [file gh-20-1-1487-s1.pdf]

Table S1. Baseline characteristics of participants for the outcome of hypertension

Variables	Under weight (n=1640)	Normal weight (n=2981)	Overweight (n=579)	Obesity (n=142)	P
BMI	17.20[16.02,17.80]	20.76[19.59,22.19]	25.15[24.45,26.04]	29.38[28.55,31.21]	<0.001
Age, years	88.00[79.00,94.00]	82.00[73.00,91.00]	79.00[70.00,89.00]	75.00[69.00,87.00]	<0.001
Male	1023(62.4)	1481(49.7)	269(46.5)	76(53.5)	<0.001
Ethnic					<0.001
Han	1473(89.8)	2793(93.7)	555(95.9)	139(97.9)	
Hui	6(0.4)	7(0.2)	3(0.5)	1(0.7)	
Zhuang	129(7.9)	93(3.1)	11(1.9)	1(0.7)	
Yao	11(0.7)	16(0.5)	1(0.2)	0(0.0)	
Korea	9(0.5)	15(0.5)	2(0.3)	0(0.0)	
Mongolia	0(0.0)	1(0.0)	0(0.0)	0(0.0)	
Others	12(0.7)	56(1.9)	7(1.2)	1(0.7)	
Current smoking status					0.002
Yes	299(18.2)	687(23.0)	131(22.6)	28(19.7)	
No	1341(81.8)	2294(77.0)	448(77.4)	114(80.3)	
Current drinking status					<0.001
Yes	260(15.9)	703(23.6)	147(25.4)	33(23.2)	
No	1380(84.1)	2278(76.4)	432(74.6)	109(76.8)	
Current exercise status					<0.001
Yes	404(24.6)	899(30.2)	219(37.8)	54(38.0)	
No	1236(75.4)	2082(69.8)	360(62.2)	88(62.0)	
Total income of year, CNY	10000.00[3600.00,21625.00]	10000.00[4000.00,23000.00]	10000.00[5000.00,20000.00]	8000.00[4000.00,20000.00]	0.689
Current marital status					<0.001

Currently married and living with spouse	464(28.3)	1239(41.6)	291(50.3)	70(49.3)	
Separated	41(2.5)	79(2.7)	7(1.2)	2(1.4)	
Divorced	6(0.4)	3(0.1)	5(0.9)	2(1.4)	
Widowed	1114(67.9)	1634(54.8)	272(47.0)	67(47.2)	
Never married	15(0.9)	26(0.9)	4(0.7)	1(0.7)	
Sleep quality					<0.001
Very good	186(11.3)	456(15.3)	94(16.2)	33(23.2)	
Good	821(50.1)	1647(55.2)	334(57.7)	79(55.6)	
So so	439(26.8)	638(21.4)	103(17.8)	22(15.5)	
Bad	182(11.1)	220(7.4)	41(7.1)	8(5.6)	
Very bad	11(0.7)	15(0.5)	5(0.9)	0(0.0)	
Not able to answer	1(0.1)	5(0.2)	2(0.3)	0(0.0)	
Sleep duration, hours	8.00[6.00,9.00]	8.00[7.00,10.00]	8.00[7.00,10.00]	8.00[7.00,10.00]	0.004
Hypertension	635(38.7)	1446(48.5)	348(60.1)	86(60.6)	<0.001

BMI: Body mass index, CNY: Chinese Yuan.

BMI category: underweight (BMI < 18.5 kg/m²), normal weight (BMI 18.5-23.9 kg/m²), overweight (BMI 24.0-27.9 kg/m²), and obesity (BMI ≥ 28.0 kg/m²).

Table S2. Baseline characteristics of participants for the outcome of diabetes mellitus

Variables	Under weight (n=1859)	Normal weight (n=3522)	Overweight (n=774)	Obesity (n=180)	P
BMI	17.26[16.02,17.84]	20.78[19.62,22.22]	25.24[24.46,26.26]	29.88[28.89,31.62]	<0.001
Age, years	88.00[79.00,94.00]	82.00[73.00,90.00]	78.00[70.00,88.00]	75.00[68.00,86.00]	<0.001
Male	1182(63.6)	1772(50.3)	383(49.5)	108(60.0)	<0.001
Ethnic					<0.001
Han	1677(90.2)	3308(93.9)	746(96.4)	176(97.8)	
Hui	6(0.3)	10(0.3)	5(0.6)	2(1.1)	
Zhuang	142(7.6)	109(3.1)	13(1.7)	1(0.6)	
Yao	11(0.6)	18(0.5)	1(0.1)	0(0.0)	
Korea	10(0.5)	16(0.5)	2(0.3)	0(0.0)	
Mongolia	0(0.0)	1(0.0)	0(0.0)	0(0.0)	
Others	13(0.7)	60(1.7)	7(0.9)	1(0.6)	
Current smoking status					<0.001
Yes	318(17.1)	781(22.2)	167(21.6)	25(13.9)	
No	1541(82.9)	2741(77.8)	607(78.4)	155(86.1)	
Current drinking status					<0.001
Yes	283(15.2)	784(22.3)	177(22.9)	34(18.9)	
No	1576(84.8)	2738(77.7)	597(77.1)	146(81.1)	
Current exercise status					<0.001
Yes	463(24.9)	1076(30.6)	309(39.9)	70(38.9)	
No	1396(75.1)	2446(69.4)	465(60.1)	110(61.1)	
Total income of year, CNY	10000.00[3500.00,23000.00]	10000.00[4000.00,24000.00]	10000.00[5000.00,24750.00]	10000.00[4000.00,25750.00]	0.175
Current marital status					<0.001

Currently married and living with spouse	521(28.0)	1471(41.8)	398(51.4)	95(52.8)	
Separated	44(2.4)	88(2.5)	12(1.6)	1(0.6)	
Divorced	6(0.3)	4(0.1)	6(0.8)	3(1.7)	
Widowed	1272(68.4)	1928(54.7)	352(45.5)	80(44.4)	
Never married	16(0.9)	31(0.9)	6(0.8)	1(0.6)	
Sleep quality					<0.001
Very good	209(11.2)	522(14.8)	123(15.9)	33(18.3)	
Good	919(49.4)	1905(54.1)	429(55.4)	102(56.7)	
So so	501(26.9)	785(22.3)	147(19.0)	30(16.7)	
Bad	213(11.5)	282(8.0)	66(8.5)	14(7.8)	
Very bad	16(0.9)	22(0.6)	7(0.9)	1(0.6)	
Not able to answer	1(0.1)	6(0.2)	2(0.3)	0(0.0)	
Sleep duration, hours	8.00[6.00,9.00]	8.00[6.00,10.00]	8.00[7.00,10.00]	8.00[7.00,10.00]	0.104
Diabetes mellitus	303(16.3)	758(21.5)	236(30.5)	57(31.7)	<0.001

BMI: Body mass index, CNY: Chinese Yuan.

BMI category: underweight (BMI < 18.5 kg/m²), normal weight (BMI 18.5-23.9 kg/m²), overweight (BMI 24.0-27.9 kg/m²), and obesity (BMI ≥ 28.0 kg/m²).

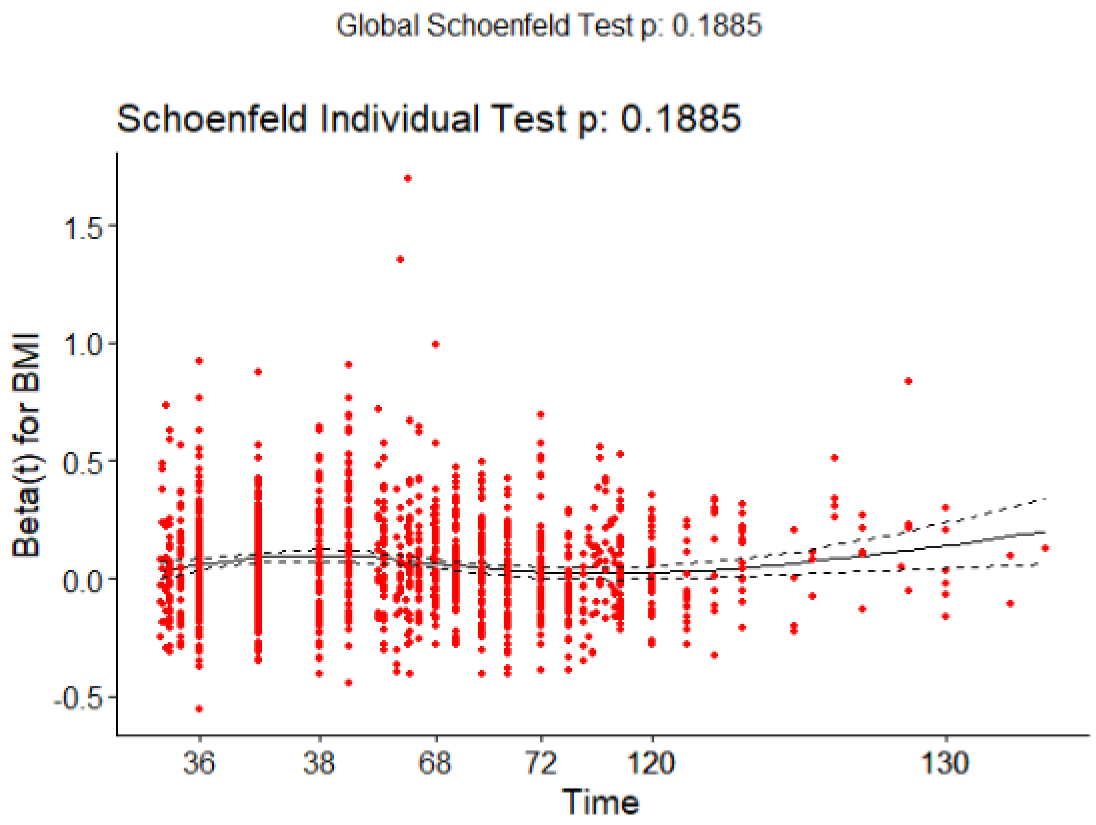

Figure S1. Schoenenfeld Individual Test for BMI as continuous variable in patients with hypertension-diabetes comorbidity.

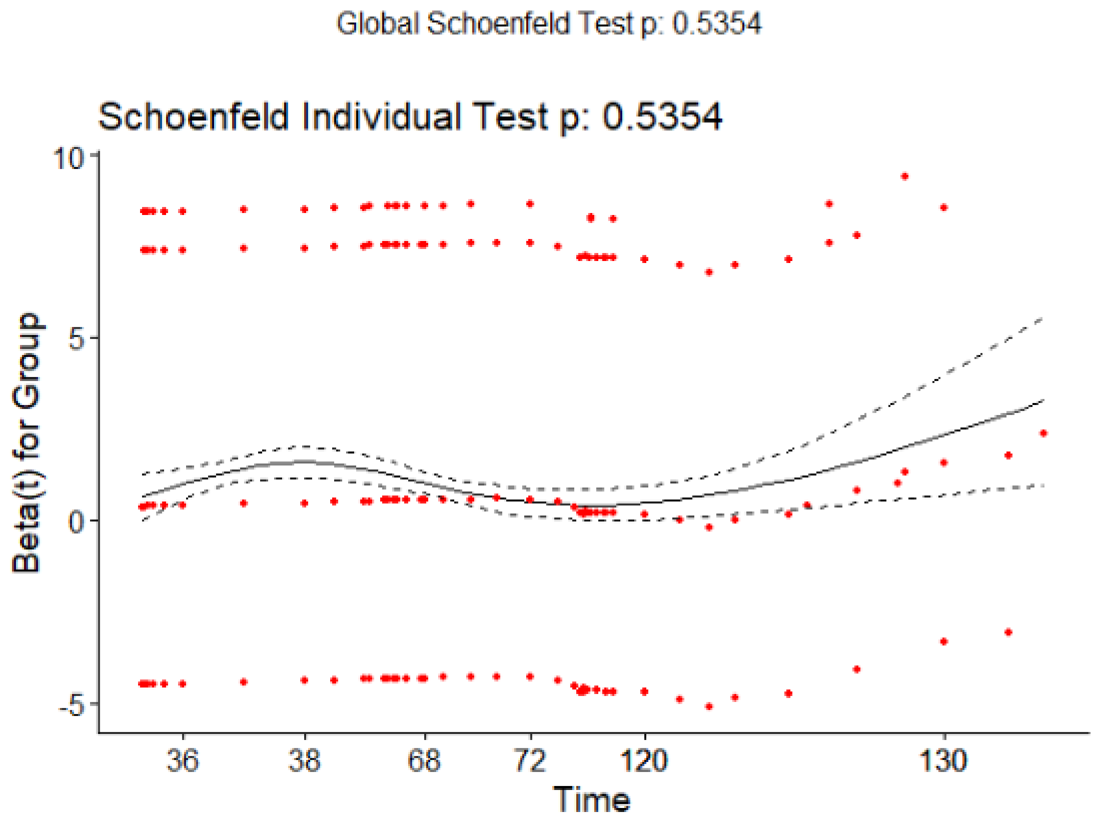

Figure S2. Schoenenfeld Individual Test for BMI group as categorical variable in patients with hypertension-diabetes comorbidity.

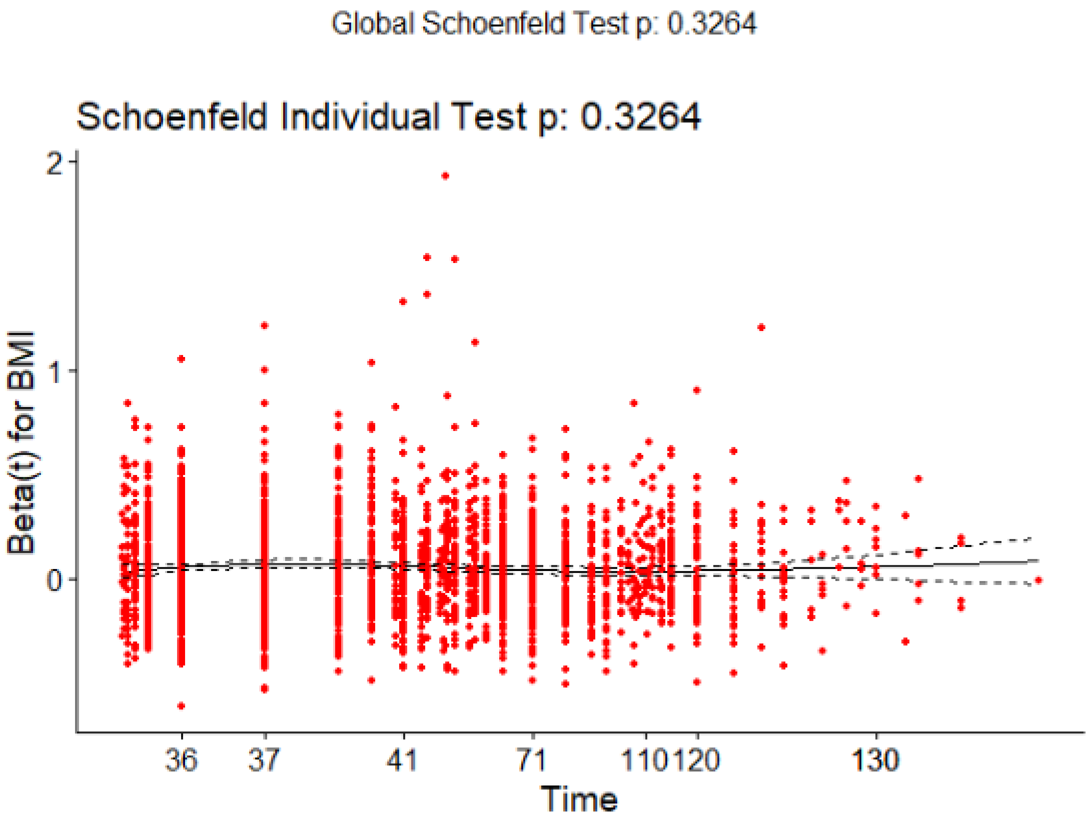

Figure S3. Schoenenfeld Individual Test for BMI as continuous variable in patients with hypertension.

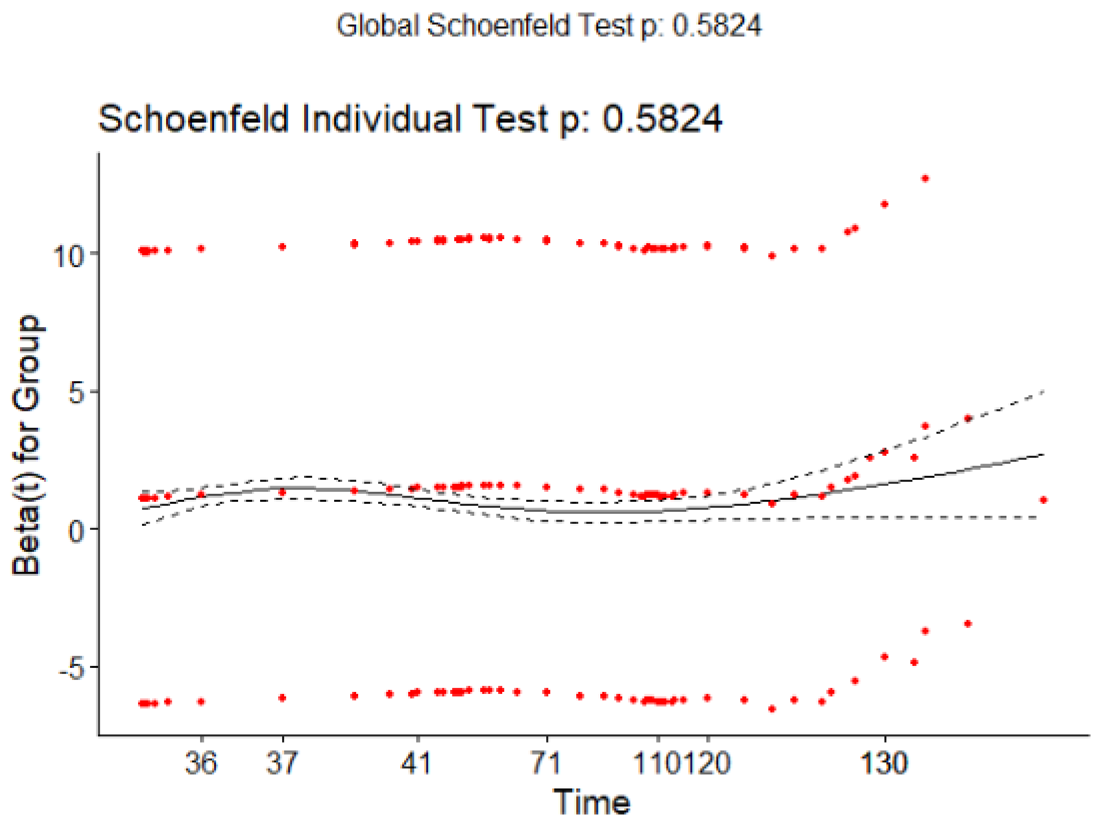

Figure S4. Schoenenfeld Individual Test for BMI group as categorical variable in patients with hypertension.

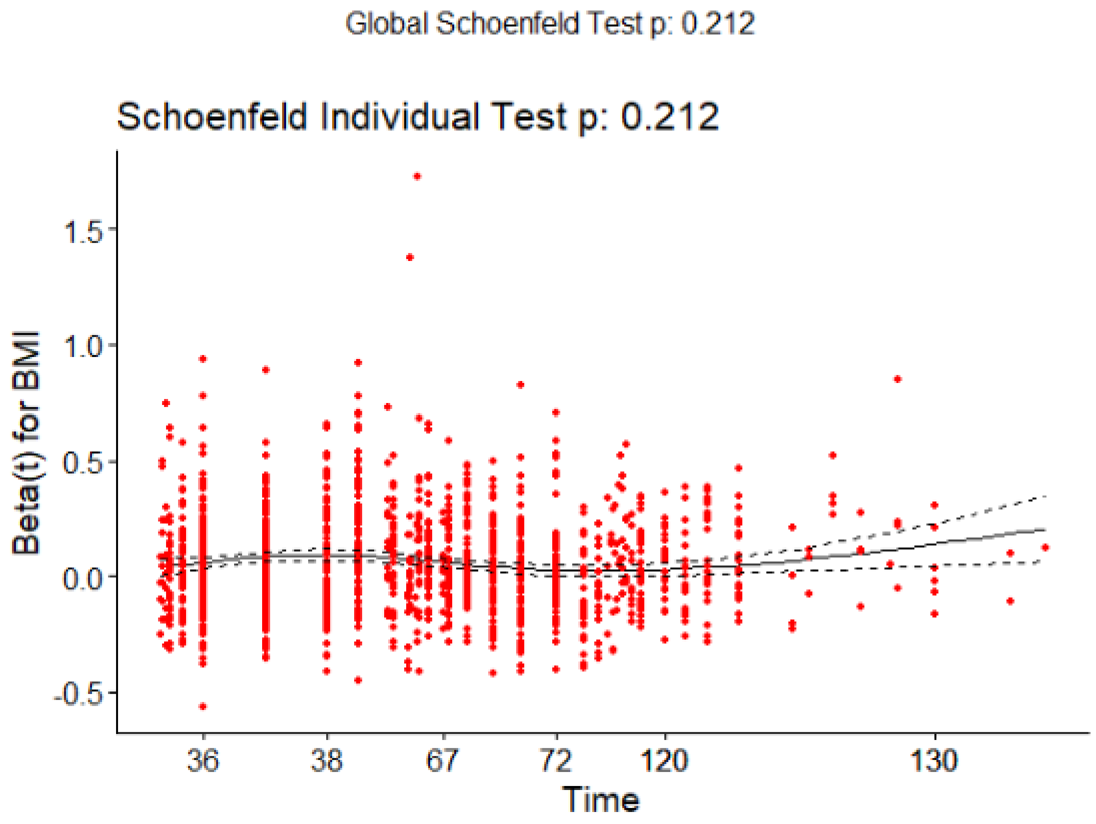

Figure S5. Schoenenfeld Individual Test for BMI as continuous variable in patients with diabetes comorbidity.

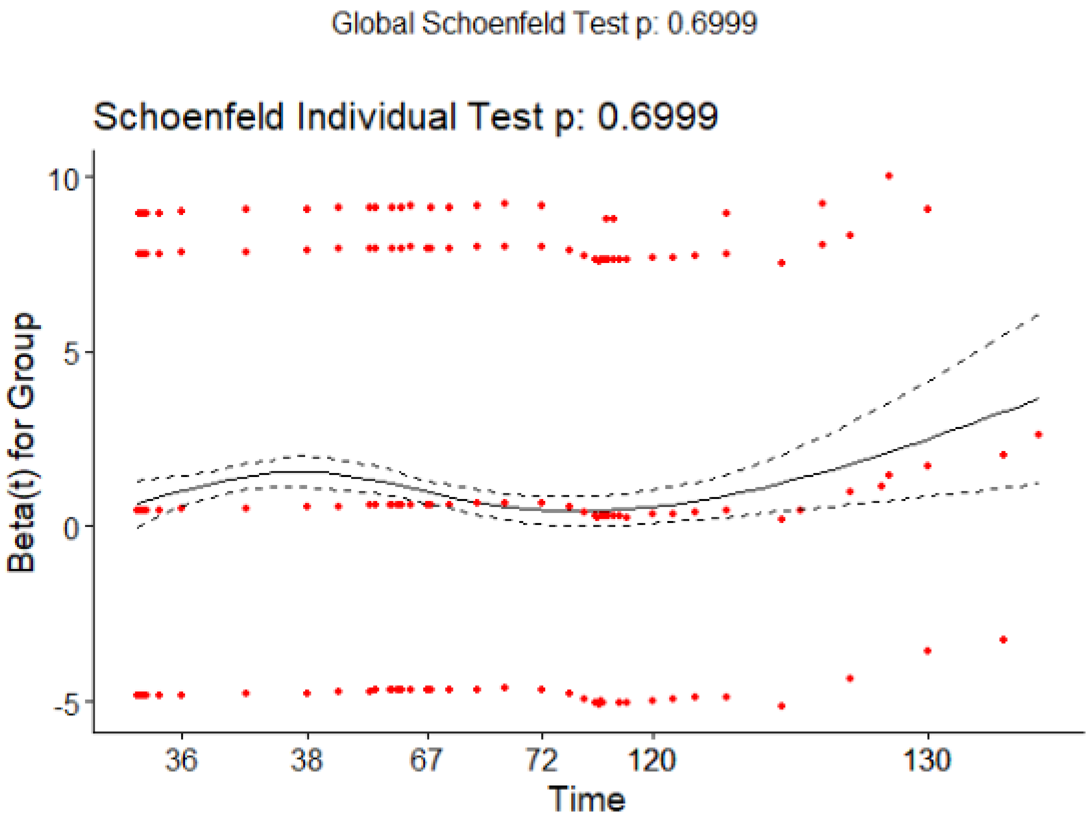

Figure S6. Schoenenfeld Individual Test for BMI group as categorical variable in patients with diabetes comorbidity.

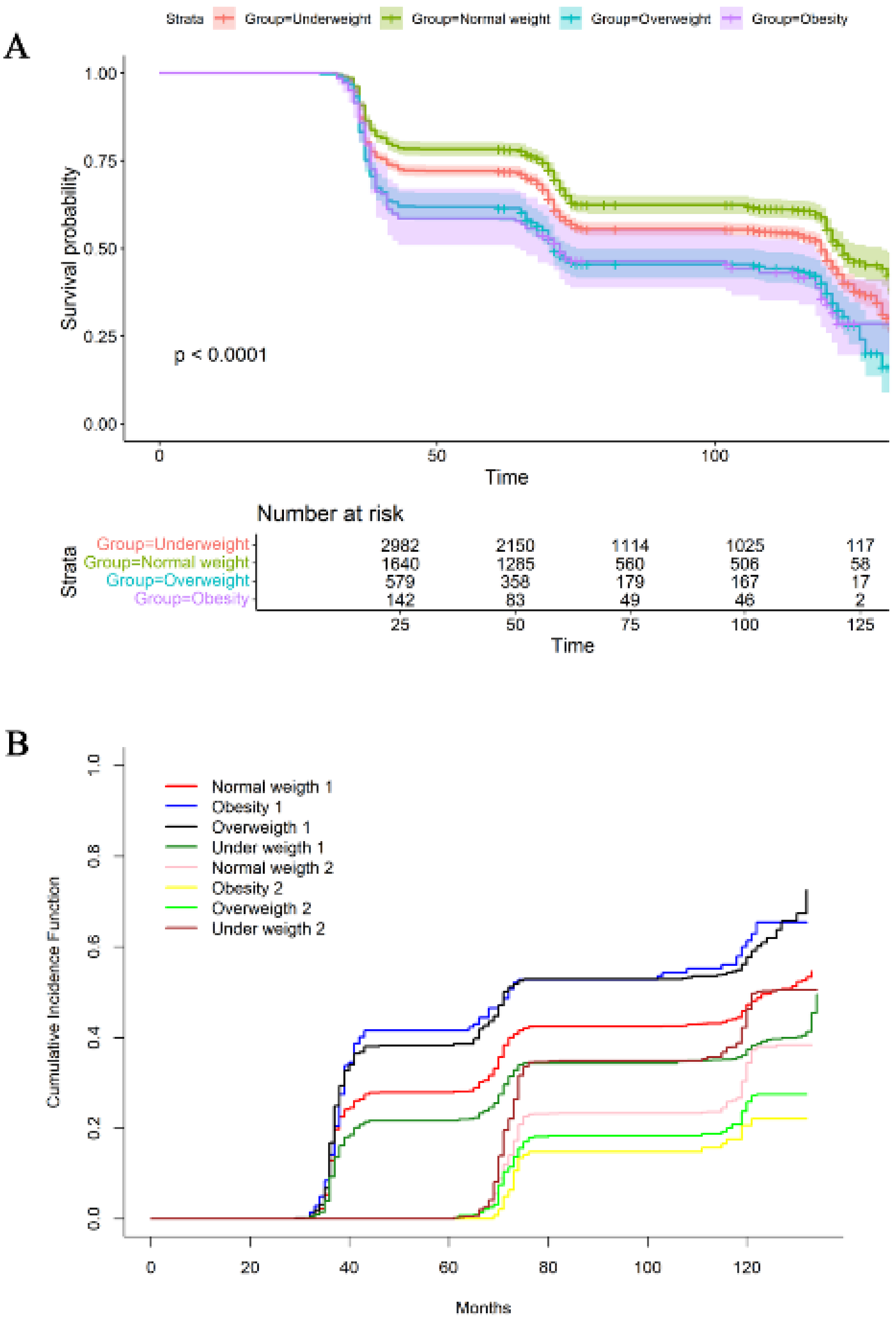

Figure S7. Survival analysis (A) and competing risks analysis regarding death (B) were performed for hypertension.

Participants were categorized into four groups based on BMI: underweight (BMI < 18.5 kg/m²), normal weight (BMI 18.5-23.9 kg/m²), overweight (BMI 24.0-27.9 kg/m²), and obesity (BMI ≥ 28.0 kg/m²).

(A) "1" indicates the occurrence of hypertension and "2" indicates the occurrence of death.

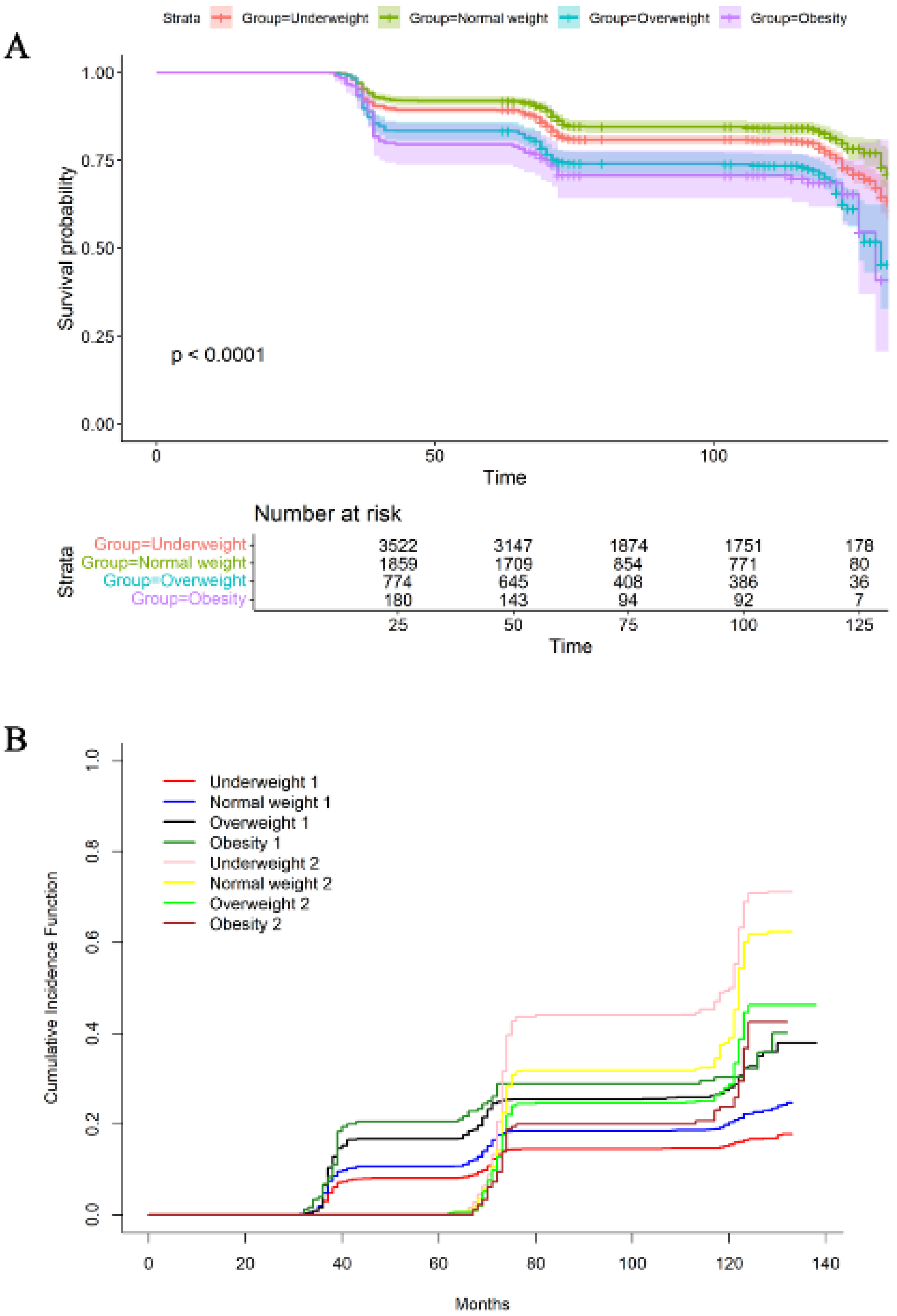

Figure S8. Survival analysis (A) and competing risks analysis regarding death (B) were performed for hypertension.

Participants were categorized into four groups based on BMI: underweight (BMI < 18.5 kg/m²), normal weight (BMI 18.5-23.9 kg/m²), overweight (BMI 24.0-27.9 kg/m²), and obesity (BMI ≥ 28.0 kg/m²).

(A) "1" indicates the occurrence of hypertension and "2" indicates the occurrence of death

Table S3. Univariate and multivariate Cox regression results for participants with hypertension as outcome.

Variables	Univariate, HR (95%CI)	P	Multivariate[a] , HR (95%CI)	P[a]	Multivariate[b] , HR (95%CI)	P[b]
BMI group						
Normal weight	1.000(Reference)		1.000(Reference)			
Underweight	1.309(1.192, 1.437)	<0.001	1.212(1.102,1.332)	<0.001		
Overweight	1.808(1.587,2.061)	<0.001	1.611(1.409,1.841)	<0.001		
Obesity	1.807(1.442,2.263)	<0.001	1.559(1.242,1.958)	<0.001		
BMI	1.052(1.041,1.062)	<0.001			1.040(1.029,1.052)	<0.001
Age, years	0.985(0.981,0.988)	<0.001	0.987(0.983,0.991)	<0.001	0.988(0.984,0.991)	<0.001
Sex						
Female	1.000(Reference)					
Male	0.966(0.894,1.045)	0.389				
Ethnic						
Han	1.000(Reference)		1.000(Reference)		1.000(Reference)	
Non-han	0.561 (0.468, 0.672)	<0.001	0.583(0.486,0.700)	<0.001	0.588(0.490,0.705)	<0.001
Current smoking status						
No	1.000(Reference)					
Yes	1.024(0.932,1.124)	0.627				
Current drinking status						
No	1.000(Reference)					
Yes	1.073(0.977,1.178)	0.140				
Current exercise status						
No	1.000(Reference)		1.000(Reference)		1.000(Reference)	
Yes	1.111(1.021,1.209)	0.015	1.037 (0.952,1.129)	0.405	1.025(0.942,1.116)	0.566
Current marital status						

Never married	1.000(Reference)	
Widowed	1.147(0.729,1.804)	0.554
Currently married and living with spouse	1.373(0.872,2.160)	0.171
Divorced	1.121(0.471,2.668)	0.795
Separated	1.349(0.812,2.239)	0.248
Total income of year, CNY	1.000(1.000,1.000)	0.135
Sleep duration, hours	1.004(0.997,1.012)	0.239
Sleep quality		
Very good	1.000(Reference)	
Good	0.960(0.855,1.077)	0.485
Bad	0.898(0.757,1.065)	0.216
So so	1.021(0.897,1.163)	0.754
Very bad	0.990(0.581,1.688)	0.970
Not able to answer	1.045(0.390,2.800)	0.930

HR: Hazard ratio, CI: Confidence interval, BMI: Body mass index, CNY: Chinese Yuan.

BMI category: underweight (BMI < 18.5 kg/m²), normal weight (BMI 18.5-23.9 kg/m²), overweight (BMI 24.0-27.9 kg/m²), and obesity (BMI ≥ 28.0 kg/m²).

[a]: In the multivariate Cox regression analysis, BMI is included in the model as a categorical variable.

[b]: In the multivariate Cox regression analysis, BMI is included in the model as a continuous variable.

Table S4. Univariate and multivariate Cox regression results for participants with diabetes mellitus as outcome

Variables	Univariate, HR (95%CI)	P	Multivariate[a] , HR (95%CI)	P[a]	Multivariate[b] , HR (95%CI)	P[b]
BMI group						
Normal weight	1.000(Reference)		1.000(Reference)			
underweight	0.752(0.659,0.860)	<0.001	0.842(0.73, 0.964)	0.013		
overweight	1.480(1.279,1.713)	<0.001	1.365(1.178,1.582)	<0.001		
obesity	1.579(1.206,2.066)	<0.001	1.404(1.071,1.841)	0.014		
BMI	1.060(1.046,1.074)	<0.001			1.042(1.027,1.057)	<0.001
Age, years	0.980(0.975,0.985)	<0.001	0.984(0.978,0.990)	<0.001	0.984(0.978,0.990)	<0.001
Sex						
Female	1.000(Reference)					
Male	0.997(0.896,1.109)	0.955				
Ethnic						
Han	1.000(Reference)		1.000(Reference)		1.000(Reference)	
Non-han	0.336(0.242,0.466)	<0.001	0.369(0.266,0.513)	<0.001	0.368(0.265,0.512)	<0.001
Current smoking status						
No	1.000(Reference)					
Yes	1.043(0.916,1.188)	0.523				
Current drinking status						
No	1.000(Reference)					
Yes	1.059(0.929,1.206)	0.393				
Current exercise status						
No	1.000(Reference)		1.000(Reference)		1.000(Reference)	
Yes	1.215(1.086,1.359)	<0.001	1.092(0.975,1.223)	0.129	1.088(0.971,1.219)	0.146

Current marital status						
Never married	1.000(Reference)		1.000(Reference)		1.000(Reference)	
Widowed	2.208(0.916,5.322)	0.078	2.991(1.237,7.232)	0.015	2.951(1.220,7.138)	0.016
Currently married and living with spouse	3.010(1.248,7.255)	0.014	2.491(0.956,6.491)	0.062	2.456(0.942,6.403)	0.066
Divorced	1.823(0.436,7.628)	0.411	1.534(0.366,6.427)	0.558	1.540(0.368,6.449)	0.554
Separated	2.032(0.783,5.278)	0.145	2.765(1.141,6.700)	0.024	2.733(1.128,6.625)	0.026
Total income of year, CNY	1.001(1.001,1.001)	0.028	1.001(1.001,1.001)	0.026	1.001(1.001,1.001)	0.032
Sleep duration, hours	1.010(1.001,1.019)	0.027	1.013(1.005,1.022)	0.002	1.013(1.005,1.022)	0.002
Sleep quality						
Very good	1.000(Reference)					
Bad	0.928(0.741,1.163)	0.519				
Good	0.925(0.790,1.083)	0.332				
So so	0.969(0.812,1.156)	0.725				
Very bad	1.138(0.635,2.038)	0.664				
Not able to answer	0.512(0.072,3.655)	0.505				

HR: Hazard ratio, CI: Confidence interval, BMI: Body mass index, CNY: Chinese Yuan.

BMI category: underweight (BMI < 18.5 kg/m²), normal weight (BMI 18.5-23.9 kg/m²), overweight (BMI 24.0-27.9 kg/m²), and obesity (BMI ≥ 28.0 kg/m²).

[a]: In the multivariate Cox regression analysis, BMI is included in the model as a categorical variable.

[b]: In the multivariate Cox regression analysis, BMI is included in the model as a continuous variable.

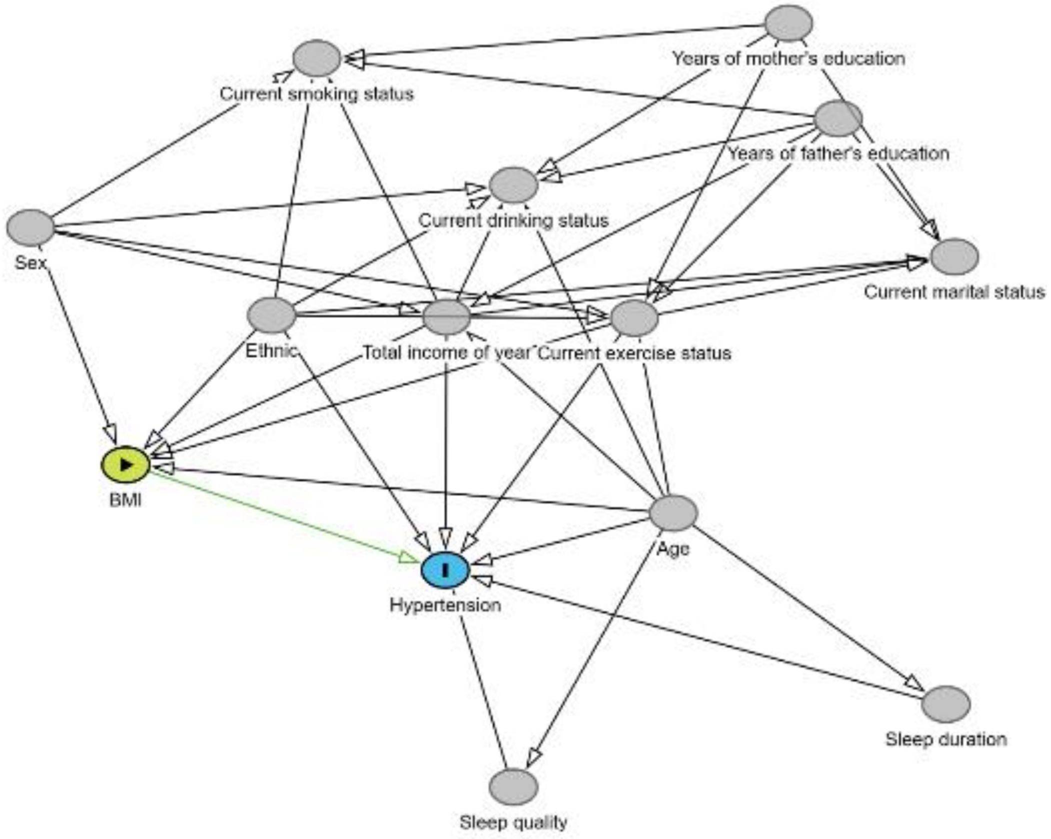

Figure S9. Directed Acyclic Graph (DAG) with Body Mass Index (BMI) as the exposure and hypertension as the outcome.

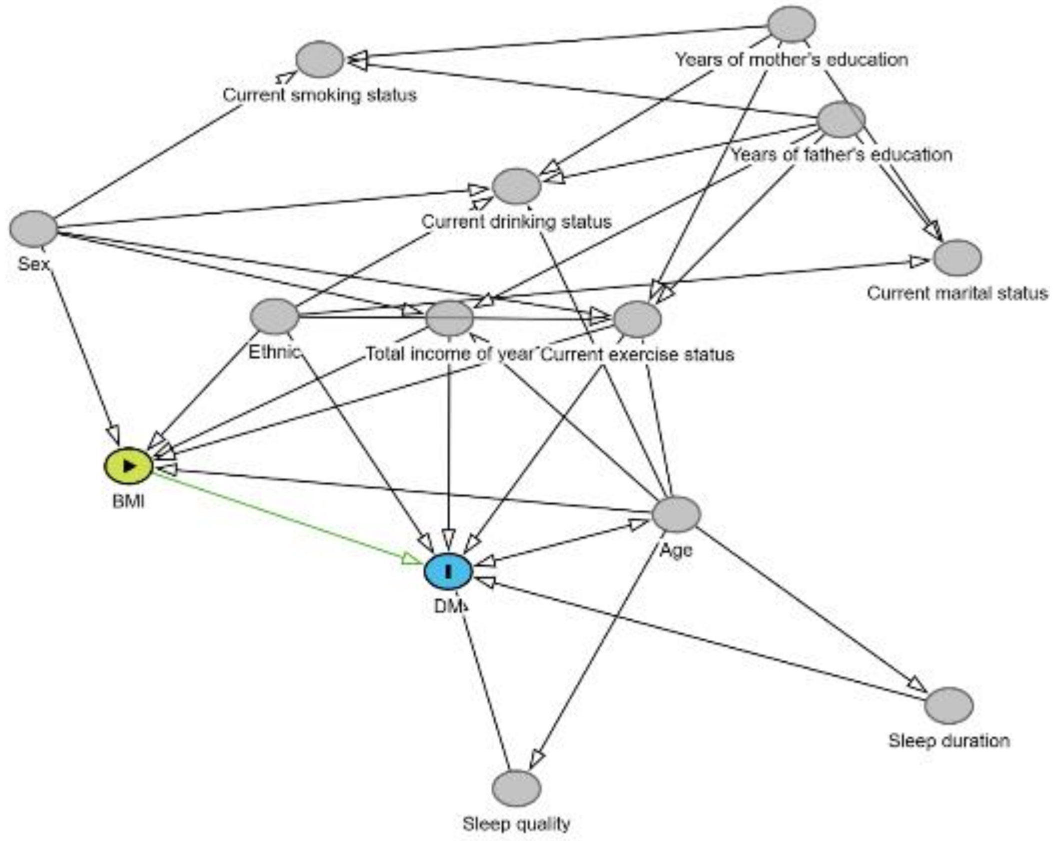

Figure S10. Directed Acyclic Graph (DAG) with Body Mass Index (BMI) as the exposure and diabetes mellitus (DM) as the outcome.

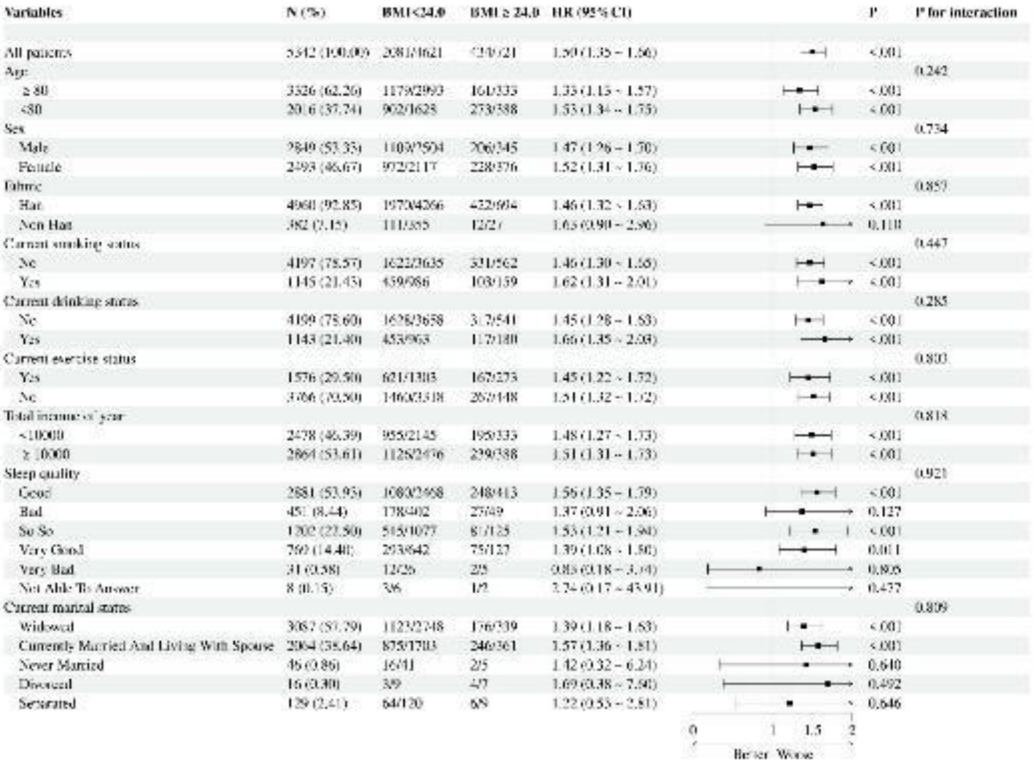

Figure S11. Subgroup analysis for hypertension with body mass index (BMI) as the exposure.

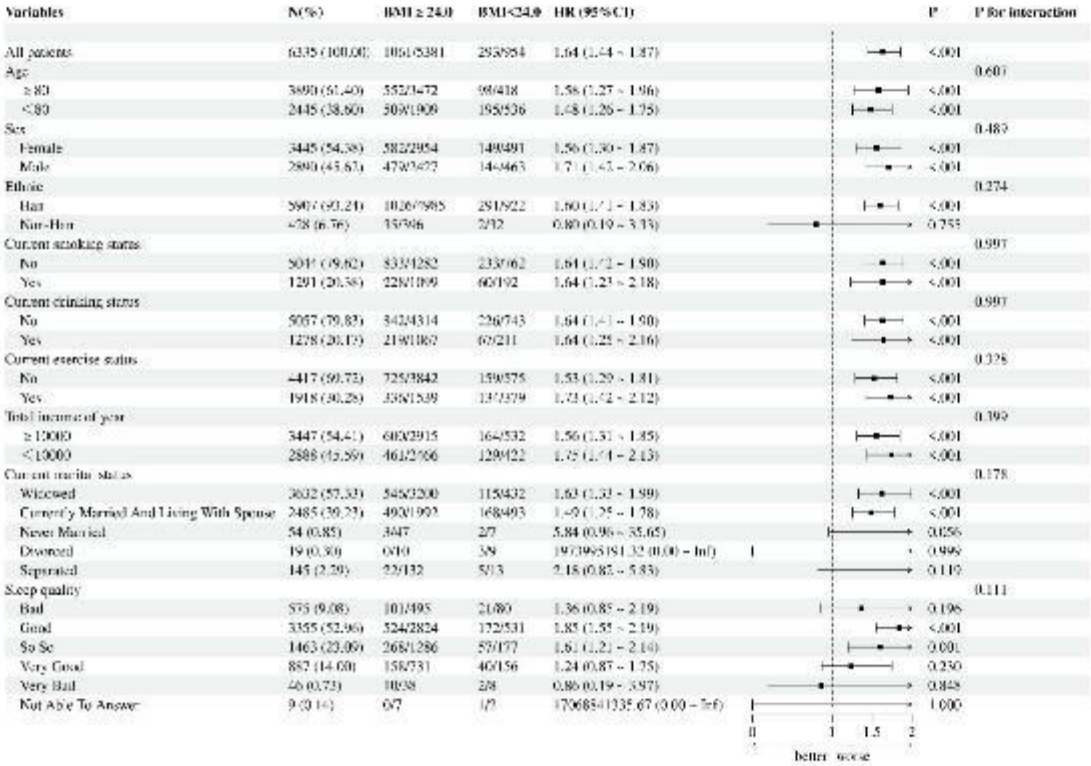

Figure S12. Subgroup analysis for diabetes mellitus with body mass index (BMI) as the exposure.
